# Exosomal Preconditioning of Human iPSC-Derived Cardiomyocytes Beneficially Alters Cardiac Electrophysiology and Micro RNA Expression

**DOI:** 10.3390/ijms25158460

**Published:** 2024-08-02

**Authors:** Øystein Røsand, Jianxiang Wang, Nathan Scrimgeour, Gurdeep Marwarha, Morten Andre Høydal

**Affiliations:** Group of Molecular and Cellular Cardiology, Department of Circulation and Medical Imaging, Faculty of Medicine and Health, Norwegian University of Science and Technology (NTNU), 7030 Trondheim, Norway; oystein.rosand@ntnu.no (Ø.R.); jianxiang.wang@ntnu.no (J.W.); nathan.scrimgeour@ntnu.no (N.S.); gurdeep.marwarha@ntnu.no (G.M.)

**Keywords:** electrophysiology, excitation–contraction coupling, exosomes, hypoxia, next-generation sequencing, micro RNA modulation

## Abstract

Experimental evidence, both in vitro and in vivo, has indicated cardioprotective effects of extracellular vesicles (EVs) derived from various cell types, including induced pluripotent stem cell-derived cardiomyocytes. The biological effects of EV secretion, particularly in the context of ischemia and cardiac electrophysiology, remain to be fully explored. Therefore, the goal of this study was to unveil the effects of exosome (EXO)-mediated cell–cell signaling during hypoxia by employing a simulated preconditioning approach on human-induced pluripotent stem cell-derived cardiomyocytes (hIPSC-CMs). Electrophysiological activity of hIPSC-CMs was measured using a multielectrode array (MEA) system. A total of 16 h of hypoxic stress drastically increased the beat period. Moreover, hIPSC-CMs preconditioned with EXOs displayed significantly longer beat periods compared with non-treated cells after 16 h of hypoxia (+15.7%, *p* < 0.05). Furthermore, preconditioning with hypoxic EXOs resulted in faster excitation–contraction (EC) coupling compared with non-treated hIPSC-CMs after 16 h of hypoxia (−25.3%, *p* < 0.05). Additionally, microRNA (miR) sequencing and gene target prediction analysis of the non-treated and pre-conditioned hIPSC-CMs identified 10 differentially regulated miRs and 44 gene targets. These results shed light on the intricate involvement of miRs, emphasizing gene targets associated with cell survival, contraction, apoptosis, reactive oxygen species (ROS) regulation, and ion channel modulation. Overall, this study demonstrates that EXOs secreted by hIPSC-CM during hypoxia beneficially alter electrophysiological properties in recipient cells exposed to hypoxic stress, which could play a crucial role in the development of targeted interventions to improve outcomes in ischemic heart conditions.

## 1. Introduction

Despite significant research efforts, ischemic heart disease (IHD) is still the leading cause of mortality worldwide [1]. IHD is primarily caused by partial or total disruption of blood flow to the myocardium, resulting in reduced delivery of oxygen and nutrients to the cardiac cells. The hypoxic stress elicited by IHD induces metabolic stress, e.g., metabolic acidosis, accumulation of reactive oxygen species (ROS), and Ca^2+^ overload, which, if not resolved, results in cardiomyocyte apoptosis and/or necrosis, and ultimately, irreversible damage of the heart. If the cardiac damage exceeds a critical limit, patients will suffer from acute myocardial infarction (AMI) [2,3,4,5]. AMI is strongly associated with dysregulation of cardiac function and alterations in electrophysiological properties. Propper cardiac function relies on a tight regulation of cardiac depolarization, contraction and relaxation, and finally repolarization [6]. This is facilitated by various molecules in cardiomyocytes, e.g., ion channels, transporters, and intracellular Ca^2+^-handling proteins [7]. Irregular cardiac electrophysiology leads to cardiac arrhythmias that can negatively impact the myocardium’s ability to pump blood [7,8].

In cardiac physiology and pathology, there has been an increased interest in researching the intracellular communication facilitated by EVs. Exosome (EXO) is the most frequently used term to describe a subgroup of EVs characterized by their 30–150 nm diameter and biogenetic origin [9,10,11]. No longer regarded as just a means for the cells to discard waste, EXOs are now understood to facilitate intercellular communication by transporting signaling molecules between donor and target cells [11]. Internalized proteins, lipids, coding and non-coding RNA, DNA, and metabolites play essential roles in intercellular communication, changing the fate of recipient cells. Among these internalized elements, microRNA (miR) are emerging as important regulators of myocardial biology and disease, as evidenced by multiple studies [12,13]. miRs can regulate gene expression through transcriptional modulation, epigenetic targeting, or post-transcriptional regulation [14,15,16,17]. It is already well established that ischemia induces profound changes in miR expression, which is important for myocardial ischemia-related genes [18]. However, new evidence has demonstrated that miRs also can regulate the electrophysiology of the heart by either conventionally affecting the gene expression of ion channels, transporters, intracellular Ca^2+^-handling proteins, and other relevant factors [19,20,21,22,23], or by direct biophysical modulation of ion channels [24].

During the last decade, considerable resources have been used to develop cardioprotective strategies based on EXOs released from human-induced pluripotent stem cell-derived cardiomyocytes (hIPSC-CMs). Both in vitro and in vivo studies have shown that treatment with EVs secreted from IPSCs enhance angiogenesis, enhance cell proliferation, reduce cardiomyocyte apoptosis, and improve left ventricular function after myocardial infarction [25,26,27]. Of note, ischemic preconditioning is once such therapeutic strategy, where EXOs isolated from ischemic cardiomyocytes, once administrated, can elicit cardioprotective effects in recipient cardiomyocytes under ischemic conditions [28,29]. However, despite these promising data, the paracrine EXO signaling between cardiomyocytes of the same myocardium, as well as EXO signaling during ischemia, remains poorly understood. Improving our knowledge of cell–cell signaling within the failing heart may give rise to therapeutic solutions in the future. Therefore, this study aimed to investigate whether EXOs released by hIPSC-CMs during hypoxia can positively influence cardiac electrophysiology and miR expression during hypoxic stress. A multielectrode array (MEA) system was used as an in vitro method of recording real-time cardiac electrophysiological activity, highlighting the biological effects facilitated by hypoxic EXOs. Additionally, miR-sequencing was performed to compare miR expressions in EXO-preconditioned and non-preconditioned hIPSC-CMs, shedding light on important modulators of cardiac electrophysiology. Lastly, an miR gene target prediction analysis was performed, bridging the MEA and miR-sequencing analysis, emphasizing possible therapeutic gene targets for future studies. Thus, this study offers novel insights into the mechanisms of intercellular communication, facilitated by EXOs, during hypoxic stress across cardiomyocytes of the same group.

## 2. Results

### 2.1. EXO Isolation

**Figure 1:**
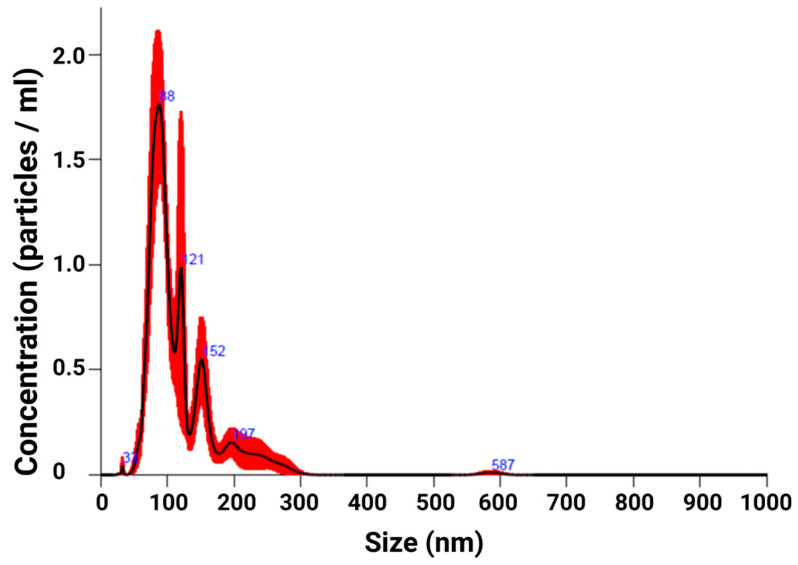
Exosomes (EXOs) were isolated with a Total Exosome Isolation kit and analyzed using a Nanosight NS300. Nanoparticle tracing analysis (NTA) analysis showed particle size and concentration distribution for EXOs secreted during hypoxia. Particle sizes were captured five times for 60 s for every sample. The mean diameter of EXOs secreted under hypoxic conditions was measured to be 167.4 nm (±25.6 nm). The mean concentration of secreted EXOs was measured to be 9.2 × 10^7^ EXO/mL (±2.0 × 10^7^ EXO/mL). Data were analyzed using the NanoSight Software NTA 3.2 Dev Build 3.2.16. Black line shows particle concentration while red error bars indicate ± SEM.

Nanoparticle tracing analysis (NTA) of isolated EXOs (Figure 1) revealed a mean particle size of 167.4 nm (±25.6 nm). Furthermore, NTA measured EXO concentrations of 9.2 × 10^7^ EXOs/mL (±2.0 × 10^7^ EXOs/mL). Our NTA analysis detected particles predominately in the 30–200 nm size range, which is consistent with previous reports of EXO size range [30]. Few to no larger particles were detected. 

### 2.2. Electrophysiological Activity of hIPSC-CMs

**Figure 2:**
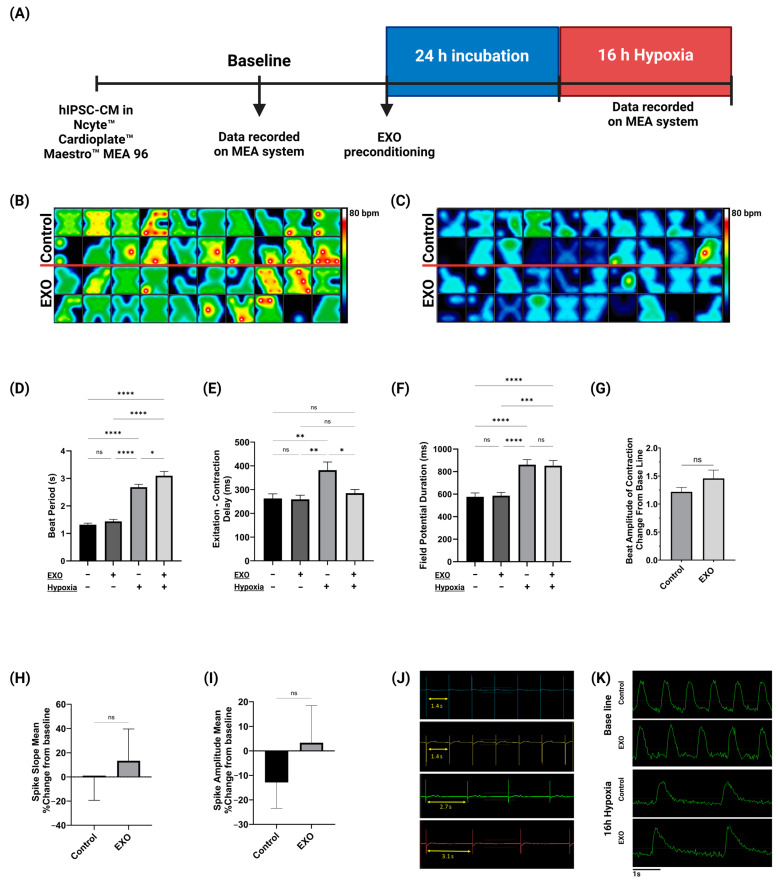
hIPSC-CM electrophysiological activity was recorded in vitro on an MEA system. (**A**) Experimental setup of the MEA recordings, EXO preconditioning, and hypoxic stimulation. A representative image of the recorded MEA activity map shows hIPSC-CM beat rate at baseline (**B**) and after 16 h of hypoxia (**C**). Saturation was set to 80 bpm. (**D**) hIPSC-CMs displayed a significant increase in beat period after 16 h of hypoxia. Furthermore, cells preconditioned with EXOs exhibited increased beat period compared with non-treated cells. (**E**) The 16 h of hypoxia significantly increased excitation–contraction (EC)-coupling for the control group. Interestingly, preconditioning with hypoxic EXOs resulted in faster EC-coupling compared with non-treated CMs after 16 h of hypoxia. (**F**) Field potential duration (FPD)for both preconditioned and non-treated hIPSC was significantly increased after 16 h of hypoxia. There were no significant changes in FPD between the two experimental groups. (**G**) EXO preconditioning did not affect the beat amplitude of contraction after 16 h of hypoxia. The were no significant changes in (**H**) FPD spike slope or (**I**) FPD spike amplitude between the control and preconditioned groups after hypoxia. (**J**) Visual representation of MEA recordings of hIPSC-CM beat period for the control group (blue) and EXO-treated group (yellow) at baseline and after 16 h of hypoxia for the control group (green) and the EXO-treated group (red). (**K**) Representative visualizations of cardiomyocyte contractions from the electrophysiological MEA recordings show hIPSC-CM contractions at baseline and after 16 h of hypoxia for both preconditioned and non-preconditioned hIPSC-CMs. Data were analyzed using one-way ANOVA multiple comparison analysis or *t*-test. Data from the MEA analysis are expressed as mean of raw values or %-change from baseline ± SEM (n = 20). Raw values were calculated from the mean recordings from 3 of the 8 electrodes at the time of baseline and post-hypoxic stimulus. * *p* ≤ 0.05; ** *p* ≤ 0.01; *** *p* ≤ 0.001; **** *p* ≤ 0.0001; ns: not significant (*p* > 0.05).

MEA data were analyzed at baseline and after 16 h of hypoxia (Figure 2A–C). Here, we report that 16 h of hypoxia drastically increased the beat period of both cell groups. Interestingly, after 16 h of hypoxia, we found that EXO-preconditioning of hIPSC-CMs increased the beat period significantly compared with the control group (+15.7%, *p* < 0.05) (Figure 2D). In addition, hypoxia significantly impacted hIPSC-CM excitation–contraction (EC)-coupling. We found that 16 h of hypoxia prolonged EC-coupling for the control group (+45.3%, *p* < 0.05), while hIPSC-CMs preconditioned with hypoxic EXOs displayed significantly faster EC-coupling compared to non-preconditioned CMs after 16 h of hypoxia (−25.3%, *p* < 0.05). The EC-coupling of EXO-preconditioned cells was comparable to the EC-coupling of cells at baseline normoxic recordings (Figure 2E). Our results also show that 16 h of hypoxia had a profound impact on the field potential duration (FPD) of hIPSC-CMs. We did, however, not find any difference in the FPD between the control and EXO-preconditioned hIPSC-CMs after hypoxia (Figure 2F). hIPSC-CM beat amplitude of contraction, measured by array-based impedance, was not significantly altered in EXO-preconditioned cells compared to non-treated cells (Figure 2G). Moreover, there were no significant changes in the spike slope (Figure 2H), nor the spike amplitude (Figure 2I) of the FPD, between the two groups after hypoxia. Data from the MEA recordings illustrate the beat periods (Figure 2J) and contractions (Figure 2K) for the hIPSC-CMs at baseline and after 16 h of hypoxia for both experimental groups.

**Figure 3:**
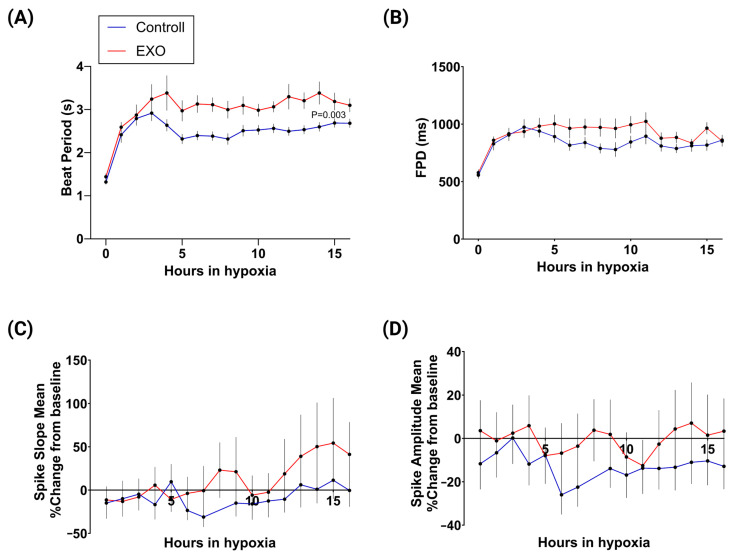
MEA recordings show electrophysiological changes during 16 h of hypoxia for control (blue) and EXO-preconditioned hIPSC-CMs (red). (**A**) The beat period was increased for both groups of hIPSC-CMs shortly after initiation and throughout the 16 h of hypoxic stress. (**B**) FPD increased for both control and preconditioned hIPSC-CMs during hypoxia. FPD was significantly increased in cells preconditioned with EXOs at 8 h of hypoxia compared to non-treated cells. Continuous MEA recordings showed no significant differences in the spike slope (**C**) or spike amplitude (**D**) of FPD between the control and preconditioned groups throughout the 16 h of hypoxia. Data were analyzed using mixed-effects analysis and Šídák’s multiple comparisons test. Data from the MEA analysis are expressed as mean of raw values or %-change from baseline ± SEM (n = 20). Raw values were calculated from the mean recordings from 3 of the 8 electrodes once every hour during the 16 h of hypoxic stimulus.

Hourly MEA recordings over the 16 h hypoxia period revealed dynamic changes in the electrophysiological activity of hIPSC-CMs under hypoxic stress (Figure 3). Our results show that the beat period for preconditioned cells was significantly longer than the non-treated cells throughout the majority of the hypoxia period (Figure 3A, *p* = 0.003). Moreover, both the beat period and the FPD showed a swift increase during the initial hours of hypoxia (1–3 h), followed by a period of stabilization. Overall, there were no significant changes in FPD between preconditioned and non-preconditioned hIPSC-CMs (Figure 3B). However, the FPD of preconditioned cells was significantly increased at 8 h of hypoxia (+31%, *p* < 0.05) compared to non-preconditioned cells. We did not observe any difference in FPD spike slope (Figure 3C) or FPD spike amplitude (Figure 3D) throughout the 16 h of hypoxia.

**Figure 4:**
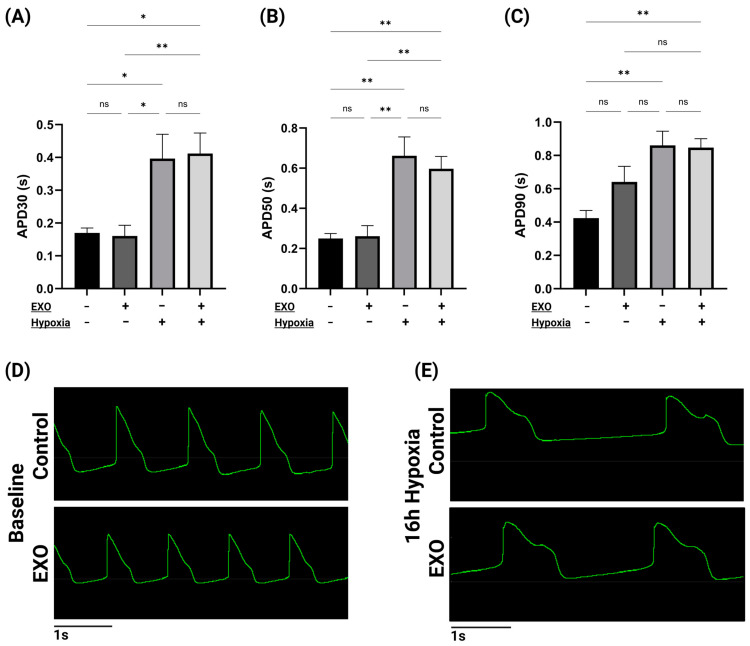
Time to membrane re-polarization extracted from local extracellular action potential (LEAP) data before and after 16 h of hypoxia. (**A**) APD30 is significantly increased after 16 h of hypoxia for both cell groups. However, EXO preconditioning did not significantly alter APD30. (**B**) The 16 h of hypoxia did significantly increase APD50 for both groups. Preconditioned hIPSC-CMs exhibited no significant change in APD50 compared with non-treated hIPSC-CMs. (**C**) The 16 h of hypoxia significantly increased APD90 for non-treated hIPSC-CMs but not for EXO-preconditioned cells. Furthermore, EXO preconditioning did not significantly alter ADP90. Representative traces from the MEA recordings show hIPSC-CM LEAP at baseline (**D**) and after 16 h of hypoxia (**E**) for both preconditioned and non-preconditioned hIPSC-CMs. Data were analyzed using one-way ANOVA multiple-comparisons analysis. Data from the MEA analysis are expressed as mean of raw values ± SEM (n = 6–8). Raw values were calculated from the mean recordings from 3 of the 8 electrodes at the time of baseline and post-hypoxic stimulus. * *p* ≤ 0.05; ** *p* ≤ 0.01; ns: not significant (*p* > 0.05).

Local extracellular action potential (LEAP) recordings (Figure 4) indicate that both APD30 and APD50 were significantly increased for both cell groups after 16 h of hypoxia (Figure 4A,B). Interestingly, APD90 was only significantly changed in non-treated cells after hypoxia (Figure 4C). Furthermore, EXO preconditioning did not significantly affect the APD30, APD50, or APD90 of hIPSC-CMs compared to the control group after 16 h of hypoxia. Data from the MEA recordings illustrate the LEAP for the hIPSC-CMs at baseline (Figure 4D) and after 16 h of hypoxia (Figure 4E) for both experimental groups.

### 2.3. miR Sequencing of Non-Treated and Pre-Conditioned hIPSC-CMs

In our study, small RNA-seq identified 1027 unique miRs in the hIPSC-CM (Table S2). Out of the detected miRs, 10 were differently expressed between the preconditioned and non-preconditioned cells. Compared with the control group, six miRs were downregulated and four miRs were upregulated in the EXO-preconditioned hIPSC-CMs (Table 1). A *p*-adjusted value of ≤0.2 was selected to ensure that only statistically significant miR regulations were included in the following target prediction analysis.

### 2.4. miR Target Prediction Analysis

**Figure 5:**
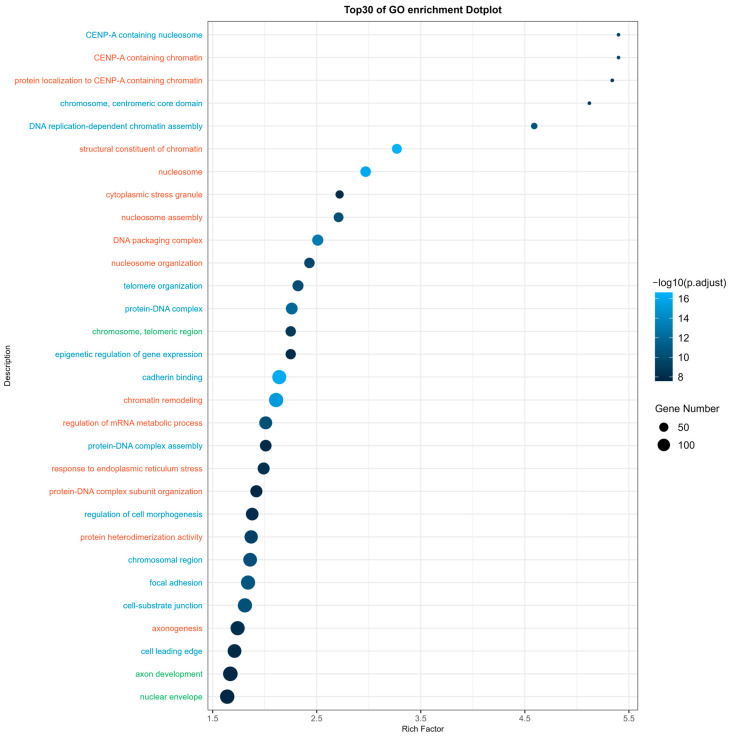
The Gene Ontology (GO) enrichment dot plot depicts the top 30 enriched GO terms for all significantly expressed miRNAs identified in the results. The *Y*-axis represents enriched terms categorized as biological process (colored in orange), cellular component (colored in blue), and molecular function (colored in green). Terms are ranked based on *p*-adjusted value. The *X*-axis denotes the Rich factor for each term. The Rich factor is defined as the ratio of input genes annotated in a term to all genes annotated in the same term. A higher Rich factor indicates a greater degree of enrichment. The size of the dot indicates the number of genes enriched in each term. The color represents the statistical significance of the enrichment, with a brighter shade of color denoting greater significance.

Here, the Gene Ontology (GO) enrichment analysis (Figure 5) revealed that the top 30 enriched GO terms correlated with significantly expressed miRs identified in the study. These terms fell into three principal domains: biological process (orange), cellular component (blue), and molecular function (green). The significance of each term was determined by its *p*-adjusted value and Rich factor, underscoring the relevance and robustness of the enrichment within the dataset. The Rich factor reflects the ratio of input genes annotated in a term compared to all genes similarly annotated, whereby a higher Rich factor suggests a more substantial enrichment. In summary, the enriched terms predominantly pertain to the umbrella concept of genetic and epigenetic information processing, cell protein interaction, macromolecule aggregation, cell morphological changes, and cell stress response—notably, the response to endoplasmic reticulum stress.

**Figure 6:**
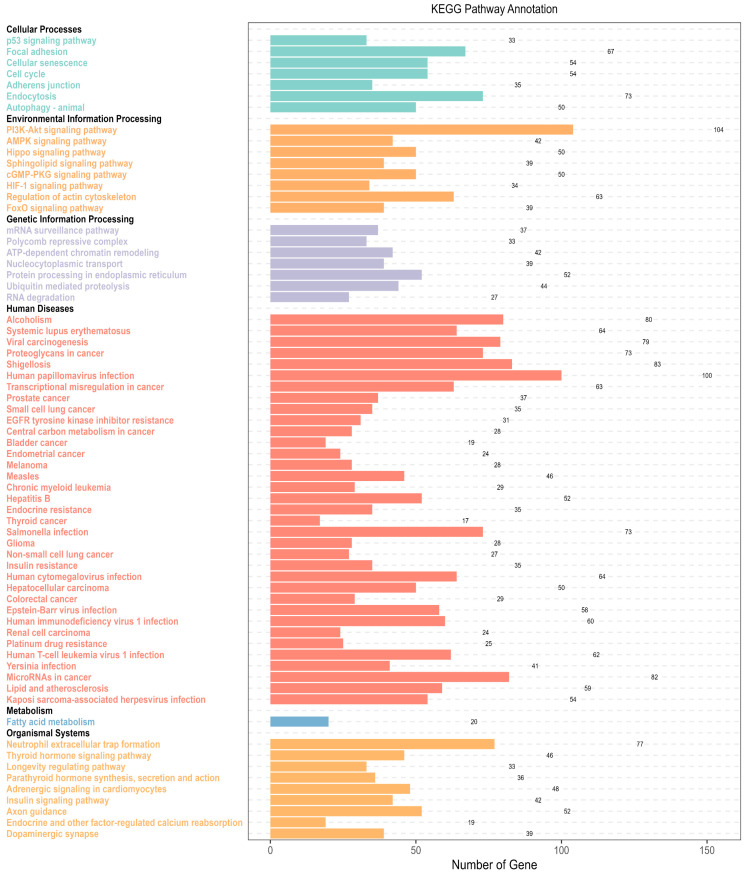
The Kyoto Encyclopedia of Genes and Genomes (KEGG) pathway annotation plot depicts the terms that are significantly enriched in various cell pathways, as identified in the KEGG database. The enriched terms are displayed along the *Y*-axis, with the BRITE hierarchy parent term in bold black and subordinate terms in various colors. A bar following each term indicates the number of genes enriched in each KEGG term, with the *X*-axis indicating the number of genes enriched in each term.

The Kyoto Encyclopedia of Genes and Genomes (KEGG) pathway annotation (Figure 6) demonstrates the significant enrichment of various cell pathways based on the KEGG database. The enriched terms fall within the BRITE hierarchy parent categories: cellular processes, environmental information processing, genetic information processing, human diseases, metabolism, and organismal systems. The interrelations among the KEGG pathways mirror the effects of the enriched terms observed in GO analysis across various aspects.

These initial analyses yielded more than 900 predicted target genes for the miRs in question (Table S3). Following the comprehensive screening process, all predicted target genes from hsa-miR-1234-3p, hsa-miR-1293, and hsa-miR-9718 were excluded due to irrelevant target function and/or no recorded tissue expression in adult CMs. Finally, 44 predicted genes targeted were identified (Table 2).

**Figure 7:**
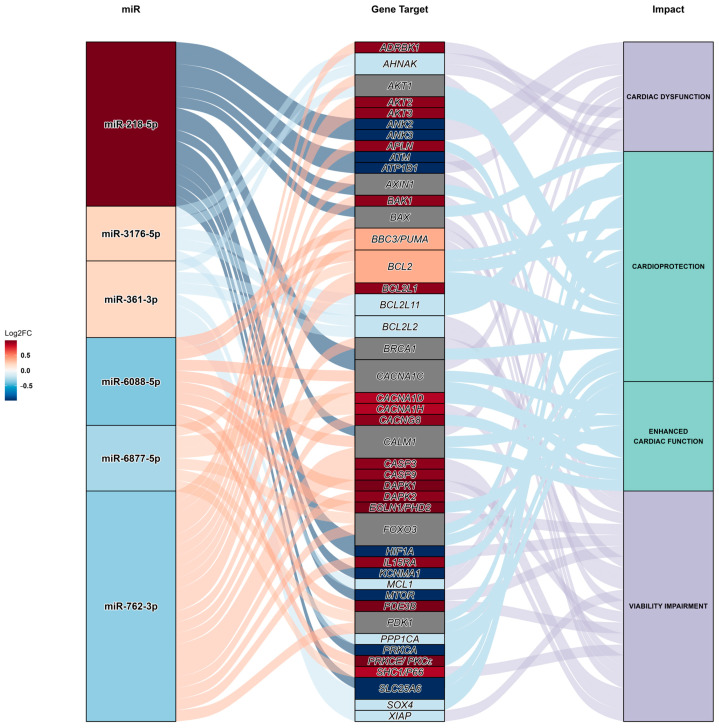
The Sankey diagram illustrates the differential expression of various miRs, regulating their predicted gene targets and the subsequent possible impact on cells, as interpretated in the experiment setting. The diagram comprises three columns with flow paths connecting various strata of the columns. The first column contains miR names, with tile colors indicating expression levels: red for upregulation and blue for downregulation. The flow ribbon color between the miR and Gene Target columns, as well as the tiles in the Impact column, indicate the inverse effect of miR expression: blue for inhibition (blue flow and tiles) and red for promotion (red flow and tiles) of target genes. Grey tiles in the Gene Target column indicate the unknown effects of inhibition or promotion, as they are predicted to be impacted by miRs with mixed effects. The flow ribbon between the Gene Target and Impact columns, as well as the tiles in the Impact column, are colored based on the effects of gene regulation on the cell. Detrimental effects are indicated in purple, while beneficial effects are shown in green.

A Sankey diagram (Figure 7) was generated as a means to illustrate the interactions of all miRs and the 44 predicted target genes. The Sankey diagram is organized into three columns representing up- and downregulated miRs, gene targets, and the impact of gene regulation. The diagram features interconnected flow paths that link different strata of the columns. The Sankey diagram shows that the upregulated mir-218-5p and downregulated mir-762-3p were predicted to target the highest number of genes. Based on the middle column of the diagram, 20 of the predicted targeted genes were predicted to be upregulated (red), while 16 were predicted to be downregulated (blue). The remaining eight predicted gene targets were predicted to be targeted by both up- and downregulated miRs. Their expression could therefore not be predicted. Furthermore, there was an even spread between possible beneficial and detrimental effects, based on the predicted targeted genes and their reported functions in CMs. Cardioprotection and viability impairment represent the largest parts of the impact column.

## 3. Discussion

Recent advances within the cardiovascular field have highlighted the potential of circulating EVs for cardioprotection. Studies have demonstrated that EVs can aid in cardiac remodeling, the enhancement of cardiac function, and the attenuation of cardiomyocyte cell death following myocardial infarction [25,26,27]. However, the biological implications of EV secretion and its effects on recipient cardiomyocytes, particularly in paracrine signaling, modulation of cardiac electrophysiological function, and miR expression during ischemic events, remain largely unexplored. The present study corroborates the profound impact of hypoxia on cardiomyocyte functionality and electrophysiological parameters. Moreover, our results show that EXOs secreted during 16 h of hypoxia beneficially alter the electrophysiological properties of hIPSC-CM, prolonging the cardiac beat period and improving EC-coupling during hypoxic stress. Next-generation sequencing identified 10 miRs differentially regulated in preconditioned cells compared to controls. Gene target prediction analysis revealed that these miRs are associated with stress response, genetic and epigenetic information processing, cell morphological changes, and cell survival. Furthermore, a total of 44 relevant gene targets were predicted to be impacted by the up- and downregulated miRs.

Our MEA data show that 16 h of hypoxia drastically increased the beat period compared to normoxic conditions. hIPSC-CM preconditioned with EXOs displayed a significantly longer beat period compared with non-treated cells after hypoxia, possibly due to the downregulation of the cyclic nucleotide gated 4 (*HCN4*) channel, known to be linked to slow heart rate [74]. A decrease in heart rate can be beneficial for CMs, as it decreases myocardial oxygen consumption and reduces the workload on the CMs, which may be advantageous in the context of hypoxia [75].

We also demonstrated that the hIPSC-CMs preconditioned with EXOs exhibited faster EC-coupling compared to non-treated cells, indicating more efficient translation of electrical signals (excitation) into mechanical contractions of the actin–myosin filaments (contraction) [76]. Cardiomyocyte contraction is regulated by complex pathways, including Ca^2+^ handling and troponin activation [77]. Improved EC-coupling without affecting the beat amplitude of contraction suggests that EXO-preconditioning influenced the rate, but not the amount, of Ca^2+^ release from the sarcoplasmic reticulum (SR). Multiple predicted gene targets of our differentially expressed miRs were predicted to impact intracellular Ca^2+^ handling. Dai et al. [37] showed that apelin (*APLN*) increased contractility in failing rat cardiac muscle by increasing Ca^2+^ availability. We found *APLN* to be a target of the downregulated mir-762-3p. Furthermore, ion channels, key modulators of Ca^2+^ regulation in cardiomyocytes, can significantly affect cardiac function. The L-type Ca^2+^ channel (LTCC) is crucial for the influx of Ca^2+^ across the sarcolemma. We predicted that several of the differentially expressed miRs target one or more subunits of the LTCC, such as the downregulated mir-762-3p, which was predicted to affect the calcium voltage-gated channel auxiliary subunit gamma 8 (*CACNG8*). *CACNG8* has been shown to encode a subunit of LTCC that is involved in regulating LTCC gating properties [51].

Despite great changes in FPD after 16 h of hypoxia, no difference in FPD, spike slope, or spike amplitude was observed between the preconditioned cells and the control cells. We found that hypoxic stress increased APD30, APD50, and APD90 in both groups. However, EXO preconditioning did not have any effect on these parameters, suggesting that cardiomyocyte membrane re-polarization was not affected by the EXO treatment. Additionally, EXO preconditioning did not significantly alter hIPSC-CM contraction amplitude after hypoxia, suggest that EXO preconditioning affected the beat rate and EC-coupling of hIPSC-CMs without greatly altering the cardiomyocyte contraction, excitability, or action potential duration of the cells.

The mitochondria are vital for intracellular Ca^2+^ dynamics via Ca^2+^ exchange, ATP generation, and ROS production. Cardiomyocytes utilize ATP to activate the contractile machinery, such as the calcium pumps (SERCA2a and the plasma membrane Ca^2+^ ATPase pump (PMCA)). ATP availability is therefore crucial for Ca^2+^ handling and cardiac function [78,79]. Furthermore, during myocardial ischemia, reducing levels of oxidative stress and inflammation helps to alleviate cellular damage. Many of our predicted gene targets were implicated in impacting ROS regulation and mitochondrial function, such as the downregulated miR-6088-5p. miR-6088-5p, which was predicted to target protein kinase C epsilon (*PRKCE*), has been shown to modulate mitochondrial function. Furthermore, upregulation of *PRKCE* has been shown to improve glucose utilization, maintain ATP production, and reduce cardiac pathophysiologic responses during chronic hypoxia [69]. Downregulation of mir-6088-5p might therefore aid the mitochondria in providing sufficient ATP to facilitate improved intracellular Ca^2+^ handling in preconditioned hIPSC-CMs [80].

Many of the miRs differentially regulated in the EXO-preconditioned hIPSC-CMs have directly been associated with cardiac disease, i.e., miR-218-5p, miR-361-3p, miR-6088-5p, and miR-762-3p. An in vitro study conducted by Sun et al. [81] found that miR-218-5p expression decreased after myocardial infarction (MI). Furthermore, they showed that miR-218-5p mediated myocardial fibrosis by inhibition of transforming growth factor beta 1 (TGF-β1). Furthermore, a loss-of-function study performed by Zhang et al. [82] showed that downregulation of miR-361-3p further progressed hypoxia-induced cell injury in H9c2 cells. Downregulated miR miR-6088-5p has previously been shown to be a possible biomarker for hypertrophic cardiomyopathy (HCM), as it was found to be downregulated in HCM patients [83]. In 2019, Yan et al. [84] published a study showing that miR-762-3p translocated in to the mitochondria and was significantly upregulated following anoxia/reoxygenation treatment. Furthermore, they also showed that myocardial ischemia/reperfusion injury was reduced by knockdown of miR-762-3p.

Accurate characterization of EXO is an important aspect of EV based research. A limitation of this study is that the characterization of the hIPSC-CM derived EXOs primarily relied on NTA. While NTA provides valuable information on particle size and concentration, it cannot reliably distinguish EXOs from other similarly sized particles, such as high-density lipoproteins (HDL). Implementing the detection of known EXO markers, such as heat shock proteins (HSP70 and HSP90), tetraspanins (CD9, CD81), or endosomal markers (TSG101), via western blotting, would enhance the robustness of the EXO characterization process [85]. However, the EXO yield from the hIPSC-CM was insufficient to meet the high sample input requirements for such analysis. Our findings should therefore be interpreted in light of this limitation.

## 4. Materials and Methods

### 4.1. hIPSC-CMs for EXO Production

For EXO production, an Ncyte™ Cardioplate™ Maestro™ MEA 96 (Ncardia, # Nc-K-NCP-M96, Leiden, The Netherlands) with pre-seeded Ncyte^TM^ hIPSC-CMs (10^4^ cells/well) was acquired and maintained following the manufacturer’s instructions. Once received, the hIPSC-CMs were maintained in Cardiomyocyte Culture Media (# Nc-M-CMCM-250, Ncardia, Leiden, The Netherlands) at 21% O_2_, 5% CO_2_ in a humidified atmosphere at 37 °C. Once settled following shipping, the cells were thoroughly washed with sterile phosphate-buffered saline (PBS) (1X) to remove any trace of the culture media before the cells were exposed to hypoxic stress: 16 h incubation with hypoxia medium (Table S1) at 1% O_2_, 5% CO_2_, 94% N_2_ in a humidified atmosphere at 37 °C [86,87]. Following 16 h of hypoxia, EXOs were isolated from the conditioned medium of the hypoxia-stressed hIPSC-CMs. hIPSC-CMs were used within 14 days of arrival, as recommended by the manufacturer.

### 4.2. EXO Isolation and Resuspension

EXO isolation was performed using Total Exosome Isolation Reagent (from the cell culture medium) (Invitrogen^TM^, #4478359, Waltham, MA, USA), following the manufacturer’s instructions. Initially, hypoxia-conditioned medium was aspirated from a 96-well MEA plate and pooled before being centrifuged at 2000× *g* for 30 min, removing cells and debris. The supernatant was then transferred to a new collection tube before a 0.5 volume of the Total Exosome Isolation Reagent (from the cell culture medium) was added. The supernatant and isolation reagent were mixed well before incubation at 2 °C to 8 °C overnight. The following day, the samples were centrifuged at 10,000× *g* for 1 h at 2 °C to 8 °C. Then, the supernatant was aspirated and discarded. Lastly, the EXO pellet was resuspended in Cardiomyocyte Culture Media. EXOs were administered as a preconditional treatment directly after isolation/resuspension.

### 4.3. EXO Quantification and Characterization

Nanoparticle Tracing Analysis (NTA) was used to ensure that hIPSC-CM-derived EXOs were sufficiently isolated. NTA was performed using a NanoSight NS300 (Malvern Instruments Ltd., Worcestershire, UK) instrument. For this purpose, EXOs secreted during hypoxic conditions were analyzed. All samples were diluted to 1:100 and the following settings were selected, following the manufacturer’s manual guide (NanoSight NS300 User Manual, MAN0541-01-EN-00, 2017): The camera level was adjusted so the particles were distinctly visible (camera level 12) and the autofocus was adjusted to avoid detecting indistinct particles. For each sample, five 60 s measurements were captured under these conditions: temperature (21 °C), syringe pump speed (70). All measurements were analyzed using the in-built NanoSight Software NTA 3.2 Dev Build 3.2.16, with the detection threshold set to 8. Hardware: camera; type: sCMOS; laser type: Blue405. To minimize data skewing from single large particles, the number of measured particles always exceeded that of the proposed minimum.

### 4.4. Culturing and Maintenance of Human IPSC-Derived Cardiomyocytes

Firstly, the Ncyte™ Cardioplate™ Maestro™ MEA 96 with pre-seeded hIPSC-CMs (10^4^ cells/well) was maintained in maintenance medium at 21% O_2_, 5% CO_2_ in a humidified atmosphere at 37 °C until stable electrophysiological activity was observed using the MEA system. Each Ncyte™ Cardioplate™ Maestro™ MEA 96 well contained 8 poly(3,4-ethylenedioxythiophere) (PEDOT) microelectrodes. A total of 20 wells were selected as a control group, and 20 wells were selected for EXO precondition. Well placement was evenly distributed for both groups to avoid skewing of the data based on the well location on the plate.

### 4.5. EXO Preconditioning

As a preconditional treatment, EXOs resuspended in Cardiomyocyte Culture Media were administered to hIPSC-CMs pre-seeded in Ncyte™ Cardioplate™ Maestro™ MEA 96, as described above. Cells were administered EXOs (200 μL/well, 9.2 × 10^4^ EXO/μL) or regular maintenance medium (200 μL/well) and the plate was incubated for 24 h at 21% O_2_, 5% CO_2_ and at 37 °C.

### 4.6. Hypoxia

Directly following EXO preconditioning, all cells were thoroughly washed with sterile PBS (1X) to remove any trace of the culture media before the cells were exposed to 16 h of hypoxic stress: incubation with hypoxia medium (200 μL/well) at 1% O_2_, 5% CO_2_, 94% N_2_ at 37 °C [87,88].

### 4.7. Analysis of Cardiac Electrophysiology on MEA System

Cells were stabilized for 10 min in the MEA system (Maestro Edge, Axion Biosystem, Atlanta, GA, USA) at 21% O_2_, 5% CO_2_ and at 37 °C. Cardiac electrophysiological activity was recorded using AxIS Navigator^TM^ version 3.9.1. Using the MEA system, field potential duration (FPD), beat period, FPD spike slope, FPD spike amplitude, excitation–contraction (EC) coupling, cardiac action potential duration (APD), beat amplitude of contraction, and local extracellular action potential (LEAP) were recorded and analyzed. LEAP induces stronger cell–electrode coupling by applying electrical signals to the electrodes. This alters the signal amplitude and shape, changing the signal from a field potential morphology to more closely resemble a cardiac action potential morphology [88]. APD30, APD50, and APD90 represent time to 30%, 50%, and 90% membrane repolarization, respectively. Data were recorded at baseline (normoxia), directly after EXO preconditioning (24 h), and directly after hypoxia (16 h). Analysis was performed using Axion software: the AxIS Metric Plotting Tool^TM^ (version 2.4) and Cardiac Analysis Tool^TM^ (version 3.3) (Axion Biosystems, Atlanta, GA, USA).

### 4.8. RNA Isolation

hIPSC-CMs exposed to hypoxic stress within the MEA system were gently washed with PBS (1X, 200 μL). Then, QIAzol lysis reagent (100 μL) (Qiagen, #79306, Hilden, Germany) was added to the cells and the lysate was collected and pooled into three samples for the control group and three samples for the EXO-preconditioned group. Subsequently, RNA was isolated using the miRNeasy Micro Kit (Qiagen, #217084, Hilden, Germany) following the manufacturer’s instructions.

### 4.9. miR Sequencing

miR sequencing was performed to evaluate the impact of the EXO preconditioning on the miR transcriptome of the hIPSC-CMs. The library preparation and sequencing service was provided by the Genomics Core Facility (GCF), Norwegian University of Science and Technology (NTNU). The GCF is funded by the Faculty of Medicine and Health Sciences at NTNU and the Central Norway Regional Health Authority. First, RNA sample quality control was performed using the RNA 6000 Pico Kit (Agilent, #5067-1513, Santa Clara, CA, USA) with a 2100 Bioanalyzer System (Agilent, #G2939BA, Santa Clara, CA, USA) following the manufacturer’s instructions. Then, a NEXTFLEX^®^ Small RNA-Seq Kit v4 with UDIs (PerkinElmer, #NOVA-5132-44 and #NOVA-5132-32, Waltham, MA, USA) was used to generate the sample library, and a 4200 TapeStation system (Agilent, Santa Clara, CA, USA) was used for quality control of the sample library. Based on the TapeStation data, sample concentrations were adjusted and then quantified using a Qubit Q32853 with the Qubit™ dsDNA BR Assay Kit (Invitrogen, #Q32850, Waltham, MA, USA). Finally, sequencing was performed on a NovaSeq 6000 (Illumina, San Diego, CA, USA) performing single reads, with 101 cycles for read 1, 10 cycles for index 1, and 10 cycles for index 2.

### 4.10. miR Target Prediction Analysis

After miR sequencing, raw data were additionally processed by the GCF. Utilizing the DESeq2 package in R [89], statistical analyses were conducted to determine the significance of miR expression differences across experimental conditions. The process involved normalizing raw count data for sequencing depth, estimating and reducing gene-wise dispersions for increased stability, and fitting a negative binomial model suitable for count data with overdispersion. The Wald test yielded a *p*-value, indicating differential expression. The Benjamini–Hochberg procedure was subsequently applied to account for the false discovery rate arising from repeated testing, yielding an adjusted *p*-value. The result of this process was the production of a datasheet consisting of 5 columns and 1028 rows. The column names were as follows: miR immature names, baseMean, log2FoldChange, lfcSE, stat, pvalue, padj (Table S2). All miRs with an adjusted *p*-value of <0.2 were selected and subjected to DIANA tool miRPath 4.0 for mature miRNA name retrieval [90]. This yielded 10 miRs. The resulting list of miRs with mature miRNA names was analyzed in R.

The list of 10 miR names was queried in three databases (miRecords, miRTarBase, and TarBase) for validated miR–target interactions using the multimiR package in R [91,92,93,94]. All unique gene targets were converted into ENTREZID using the clusterProfiler package, with 0.12% of 3450 gene names failing to map [95]. Overrepresentation analysis was performed on the resulting miR gene targets in the clusterProfiler package for Gene Ontology (GO) enrichment analysis and Kyoto Encyclopedia of Genes and Genomes (KEGG) enrichment analysis [96,97].

For the GO enrichment analysis, molecular function, biological process, and cellular component, all three of which are subcategories of GO terms in humans, were enriched from the target genes with the parameters of the *p*-value and q-value (Benjamini–Hochberg-adjusted *p*-value) set to a cut-off of 0.05. The *p*-value adjustment method was set to Benjamini–Hochberg. The results were extracted and ranked by adjusted *p*-value in a descending manner. The top 30 enriched GO terms were then plotted onto a dot plot.

KEGG Enrichment Analysis, all target genes were enriched via the clusterProfiler package with the organism set to human and the *p*-value and q-value cut-offs set to 0.05. The *p*-value adjustment method was set to Benjamini–Hochberg. Then, the BRITE hierarchy parent terms for each KEGG term were manually annotated based on the KEGG database description. All statistically significant KEGG terms were plotted onto a KEGG enrichment graph.

Based on the top terms of both GO and KEGG enrichment analyses, detailed miR-target interactions were manually examined for their relevance in the experimental context through a comprehensive screening based on target gene tissue expression (The Human Protein Atlas and GeneCards) and published studies on Web of Science and PubMed.

Lastly, a Sankey diagram was generated based on miRs, log2Fold Change, and impact columns (positive or negative) to demonstrate miR target gene effects. miRNAs with a <0.2 adjusted *p*-value were selected for the first column. Gene targets were selected for the second column, with function impact categorized into four areas: viability impairment, cardiac dysfunction, cardioprotection, and contractility/enhanced cardiac function. miRNAs were ranked based on the log2 fold changes detected, with the flow demonstrating either inhibition or promotion of the gene target and function impact.

### 4.11. Statistical Analysis

Statistical analysis of the NTA data was performed with the NanoSight Software NTA 3.2 Dev Build 3.2.16. GraphPad Prism 9 (GraphPad Software, San Diego, CA, USA) was used for statistical analyses of the MEA data. Significant differences among the samples were determined using one-way ANOVA or two-tailed independent sample *t*-test, or mixed-effects analysis: Šídák’s multiple comparisons test, depending on analysis. Quantitative data are expressed as the mean of raw values or %-change from baseline ± SEM. A minimum *p*-value of ≤0.05 was considered significant. miR target prediction analysis was conducted in R 4.3.1, utilizing several packages for over-representation analysis [98]. R packages: tidyverse [99,100], clusterProfiler [95,101], org.Hs.eg.db [100], DOSE [101], multiMiR [94,95,96,97,98,99,100,101,102]. The resulting gene targets were evaluated and ranked based on their adjusted *p*-values, with the most significant findings being selected for further interpretation in the context of the experiment.

## 5. Conclusions

This study provides new insights into vesicle-based cardioprotective strategies, specifically EXO-mediated cell–cell signaling between hypoxic hIPSC-CMs. We show that hIPSC-CMs preconditioned with hypoxic EXOs demonstrated an increased beat period and enhanced EC-coupling efficiency after hypoxic stress, suggesting potential cardioprotective benefits by reducing myocardial oxygen consumption and workload, and improving contraction cycles and overall cardiac function.

This study also highlighted the intricate involvement of miRs in these responses. Next-generation sequencing identified 10 differentially regulated miRs linked to key cardiac functions, including cell survival, contraction, apoptosis, ROS regulation, and ion channel modulation. These findings represent the potential for miR-targeted therapies in the future.

Overall, our results reveal novel aspects of EXO-mediated cardioprotection, paving the way for targeted interventions to improve outcomes in ischemic heart disease. Further studies on the identified miRs could enhance our understanding and treatment of ischemic heart disease.

## Figures and Tables

**Table 1:** Overview of significantly down- and upregulated miRs.

miR	Adjusted *p*-Value	Log2FoldChange	Regulation
miR-1234-3p	0.0001	−1.28	Down
miR-6088-5p	0.0072	−0.38	Down
miR-9718-3p	0.0110	−0.43	Down
miR-762-3p	0.0110	−0.34	Down
miR-6877-5p	0.0373	−0.29	Down
miR-1293-5p	0.0668	−0.26	Down
miR-218-5p	0.1790	0.99	Up
miR-4284-3p	0.1805	0.22	Up
miR-3176-5p	0.1805	0.22	Up
miR-361-3p	0.1997	0.21	Up

**Table 2:** Overview of predicted target genes possibly modulated by the significantly up- and downregulated miRs, and their associated functions, level of expression in human cardiomyocytes, and published studies. NA: not applicable.

Target Genes	Associated Function	Level of Expression in Human Cardiomyocytes	Author
AHNAK nucleoprotein (*AHNAK*)	Excitation–contraction coupling	Medium	[31]
AKT serine/threonine kinase 1 (*AKT1*)	Cell survival	Medium	[32]
AKT serine/threonine kinase 2 (*AKT2*)	Cell survival	Medium	[33]
AKT serine/threonine kinase 3 (*AKT3*)	Cell survival	Low	[34]
Ankyrin 2 (*ANK2*)	Ca^2+^ signaling, excitation–contraction coupling	Medium	[35]
Ankyrin 3 (*ANK3*)	Na^+^ signaling, excitability	Medium	[36]
Apelin (*APLN*)	Ca^2+^ signaling, contraction	NA	[37]
ATM serine/threonine kinase (*ATM*)	Anti-apoptotic, cardiac survival	Medium	[38]
ATPase Na^+^/K^+^ transporting subunit beta 1 (*ATP1B1*)	Contractility	Medium	[39]
Axin 1 (*AXIN1*)	Pro-apoptotic	Medium	[40]
BCL2 antagonist/killer 1 (*BAK1*)	Pro-apoptotic	Low	[41]
BCL2-associated X (*BAX*)	Pro-apoptotic	Medium	[42]
BCL2 binding component 3 (*BBC3*)	Pro-apoptotic	Medium	[43]
BCL2 apoptosis regulator (*BCL2*)	Anti-apoptotic	Medium	[44]
BCL2 Like 1 (*BCL2L1*)	Pro-apoptotic	NA	[45]
BCL2 Like 11 (*BCL2L11*)	Pro-apoptotic	Medium	[46]
BCL2-like 2 (*BCL2L2*)	Anti-apoptotic	Medium	[47]
BRCA1 DNA repair-associated (*BRCA1*)	anti-apoptotic, cell survival	Low	[48]
Calcium voltage-gated channel Subunit alpha 1C (*CACNA1C*)	L-type Ca^2+^ channel	Medium	[49]
Calcium voltage-gated channel subunit alpha 1D (*CACNA1D*)	Ca^2+^ channel	NA	[49]
Calcium voltage-gated channel subunit alpha 1H (*CACNA1H*)	ER stress response	Low	[50]
Calcium voltage-gated channel auxiliary subunit gamma 8 (*CACNG8*)	L-type Ca^2+^ channel	NA	[51]
Calmodulin 1 (*CALM1*)	Excitation–contraction coupling	Medium	[52]
Caspase 3 (*CASP3*)	Pro-apoptotic	NA	[53]
Caspase 9 (*CASP9*)	Pro-apoptotic	NA	[54]
Death-associated protein kinase 1 (*DAPK1*)	Pro-inflammatory	Medium	[55]
Death-associated protein kinase 2 (*DAPK2*)	Pro-apoptotic	Medium	[56]
Egl-9 family hypoxia inducible factor 1 (*EGLN1*)	Contractility	High	[57]
Forkhead box O3 (*FOXO3*)	Cell survival, mitochondrial function	NA	[58]
G protein-coupled receptor kinase 2 (*GRK2*)	Mitochondrial function	Low	[59]
Hypoxia inducible factor 1 subunit alpha (*HIF1A*)	Cell survival	NA	[60]
Interleukin 15 receptor subunit alpha (*IL15RA*)	Anti-apoptotic	Medium	[61]
Potassium calcium-activated channel subfamily M alpha 1 (*KCNMA1*)	Ca^2+^ channel, excitation–contraction coupling	NA	[62]
MCL1 apoptosis regulator (*MCL1*)	Anti-apoptotic	Medium	[63]
Mechanistic target of rapamycin kinase (*MTOR*)	Cell survival	High	[64]
Phosphodiesterase 3B (*PDE3B*)	Contractility	NA	[65]
Pyruvate dehydrogenase kinase 1 (*PDK1*)	Cardioprotection	Medium	[66]
Protein phosphatase 1 catalytic subunit alpha (*PPP1CA*)	Contractility	Medium	[67]
Protein kinase C Alpha (*PRKCA*)	Contractility	Medium	[68]
Protein kinase C Epsilon (*PRKCE*)	Cardioprotection	NA	[69]
SHC adaptor protein 1 (*SHC1*)	Mitochondrial function	NA	[70]
Solute carrier family 25 member 6 (*SLC25A6*)	ATP synthesis	High	[71]
SRY-box transcription factor 4 (*SOX4*)	Pro-apoptotic	Medium	[72]
X-linked inhibitor of apoptosis (*XIAP*)	Anti-apoptotic	High	[73]

## Data Availability

The datasets analyzed during this study are available from the corresponding author on reasonable request. RNA expression data can be accessed at ArrayExpress accession E-MTAB-13912: “smalRNA seq of IPSC-cardiomyocytes treated with exosomes before hypoxic stimuli”.

## Supplementary Materials

The following supporting information can be downloaded at: https://www.mdpi.com/article/10.3390/ijms25158460/s1.

## Abbreviations

AKT	AKT serine/threonine kinase
AMI	acute myocardial infarction
ANK2	ankyrin 2
ANK3	ankyrin 3
APD	action potential durations
APLN	apelin
AXIN1	axin 1
BAK1	BCL2 antagonist/killer 1
BAX	BCL2-associated X
BBC3	BCL2 binding component 3
BCL2	B-cell lymphoma 2
BCL2L1	BCL2-like 1
BCL2L11	BCL2-like 11
BCL2L2	BCL2-like 2
BRCA1	BRCA1 DNA repair associated
CACNA1C	calcium voltage-gated channel subunit alpha1 C
CACNA1D	calcium voltage-gated channel subunit alpha1 D
CACNA1H	calcium voltage-gated channel subunit alpha1 H
CACNG8	calcium voltage-gated channel auxiliary subunit gamma 8
CALM1	calmodulin 1
CASP3	caspase 3
CASP9	caspase 9
DAPK1	death-associated protein kinase 1
DAPK2	death-associated protein kinase 2
EC	excitation–contraction
EGLN1	Egl-9 family hypoxia inducible factor 1
EV	extracellular vesicles
EXO	exosome
FOXO3	forkhead box O3
FPD	field potential duration
GCF	Genomics Core Facility
GO	Gene Ontology
GRK2	G protein-coupled receptor kinase 2
HCM	hypertrophic cardiomyopathy
Hipsc-CM	human-induced pluripotent stem cell-derived cardiomyocytes
IHD	ischemic heart disease
IPSC	induced pluripotent stem cell
KEGG	Kyoto Encyclopedia of Genes and Genomes
LEAP	local extracellular action potential
LTCC	L-type Ca^2+^ channel
MCL1	MCL1 apoptosis regulator
MEA	multielectrode array
MI	myocardial infarction
miR	microRNA
MITF	microphthalmia-associated transcription factor
NTA	nanoparticle tracing analysis
NTNU	Norwegian University of Science and Technology
PEDOT	poly(3,4-ethylenedioxythiophere)
PRKCE	protein kinase C epsilon
ROS	reactive oxygen species
SHC1	SHC adaptor protein 1
SLC25A6	solute carrier family 25 member 6
SOX4	SRY-box transcription factor 4
SR	sarcoplasmic reticulum
TGF-β1	transforming growth factor beta
WHO	World Health Organization
XIAP	x-linked inhibitor of apoptosis

## References

[1] Nowbar A.N., Gitto M., Howard J.P., Francis D.P., Al-Lamee R. (2019). Mortality from ischemic heart disease: Analysis of data from the World Health Organization and coronary artery disease risk factors From NCD Risk Factor Collaboration. Circ. Cardiovasc. Qual. Outcomes.

[2] Mosterd A., Hoes A.W. (2007). Clinical epidemiology of heart failure. Heart.

[3] Greco S., Gaetano C., Martelli F. (2014). HypoxamiR regulation and function in ischemic cardiovascular diseases. Antioxid. Redox Signal..

[4] Ertracht O., Malka A., Atar S., Binah O. (2014). The mitochondria as a target for cardioprotection in acute myocardial ischemia. Pharmacol. Ther..

[5] Kalogeris T., Baines C.P., Krenz M., Korthuis R.J. (2012). Cell biology of ischemia/reperfusion injury. Int. Rev. Cell Mol. Biol..

[6] Huang C.L., Lei M. (2023). Cardiomyocyte electrophysiology and its modulation: Current views and future prospects. Philos. Trans. R. Soc. B.

[7] Yang D., Deschênes I., Fu J.D. (2022). Multilayer control of cardiac electrophysiology by microRNAs. J. Mol. Cell. Cardiol..

[8] Benjamin E.J., Muntner P., Alonso A., Bittencourt M.S., Callaway C.W., Carson A.P., Chamberlain A.M., Chang A.R., Cheng S., Das S.R. (2019). Heart disease and stroke statistics—2019 update: A report from the American Heart Association. Circulation.

[9] Kalluri R., LeBleu V.S. (2020). The biology, function, and biomedical applications of exosomes. Science.

[10] Johnstone R.M., Adam M., Hammond J., Orr L., Turbide C. (1987). Vesicle formation during reticulocyte maturation. Association of plasma membrane activities with released vesicles (exosomes). J. Biol. Chem..

[11] Mori M.A., Ludwig R.G., Garcia-Martin R., Brandão B.B., Kahn C.R. (2019). Extracellular miRNAs: From biomarkers to mediators of physiology and disease. Cell Metab..

[12] Bang C., Batkai S., Dangwal S., Gupta S.K., Foinquinos A., Holzmann A., Just A., Remke J., Zimmer K., Zeug A. (2014). Cardiac fibroblast-derived microRNA passenger strand-enriched exosomes mediate cardiomyocyte hypertrophy. J. Clin. Investig..

[13] Pegtel D.M., Cosmopoulos K., Thorley-Lawson D.A., van Eijndhoven M.A., Hopmans E.S., Lindenberg J.L., de Gruijl T.D., Würdinger T., Middeldorp J.M. (2010). Functional delivery of viral miRNAs via exosomes. Proc. Natl. Acad. Sci. USA.

[14] Hata A. (2013). Functions of microRNAs in cardiovascular biology and disease. Annu. Rev. Physiol..

[15] Barwari T., Joshi A., Mayr M. (2016). MicroRNAs in Cardiovascular Disease. J. Am. Coll. Cardiol..

[16] Kim D.H., Saetrom P., Snøve Jr O., Rossi J.J. (2008). MicroRNA-directed transcriptional gene silencing in mammalian cells. Proc. Natl. Acad. Sci. USA.

[17] Huntzinger E., Izaurralde E. (2011). Gene silencing by microRNAs: Contributions of translational repression and mRNA decay. Nat. Rev. Genet..

[18] Yu S., Li G. (2010). MicroRNA expression and function in cardiac ischemic injury. J. Cardiovasc. Transl. Res..

[19] Lu Y., Zhang Y., Wang N., Pan Z., Gao X., Zhang F., Zhang Y., Shan H., Luo X., Bai Y. (2010). MicroRNA-328 contributes to adverse electrical remodeling in atrial fibrillation. Circulation.

[20] Barana A., Matamoros M., Dolz-Gaitón P., Pérez-Hernández M., Amorós I., Núñez M., Sacristán S., Pedraz Á., Pinto Á., Fernández-Avilés F. (2014). Chronic atrial fibrillation increases microRNA-21 in human atrial myocytes decreasing L-type calcium current. Circ. Arrhythm. Electrophysiol..

[21] Luo X., Pan Z., Shan H., Xiao J., Sun X., Wang N., Lin H., Xiao L., Maguy A., Qi X.Y. (2013). MicroRNA-26 governs profibrillatory inward-rectifier potassium current changes in atrial fibrillation. J. Clin. Investig..

[22] Terentyev D., Belevych A.E., Terentyeva R., Martin M.M., Malana G.E., Kuhn D.E., Abdellatif M., Feldman D.S., Elton T.S., Györke S. (2009). miR-1 overexpression enhances Ca(2+) release and promotes cardiac arrhythmogenesis by targeting PP2A regulatory subunit B56alpha and causing CaMKII-dependent hyperphosphorylation of RyR2. Circ. Res..

[23] Drawnel F.M., Wachten D., Molkentin J.D., Maillet M., Aronsen J.M., Swift F., Sjaastad I., Liu N., Catalucci D., Mikoshiba K. (2012). Mutual antagonism between IP(3)RII and miRNA-133a regulates calcium signals and cardiac hypertrophy. J. Cell. Biol..

[24] Yang D., Wan X., Dennis A.T., Bektik E., Wang Z., Costa M.G.S., Fagnen C., Vénien-Bryan C., Xu X., Gratz D.H. (2021). MicroRNA Biophysically Modulates Cardiac Action Potential by Direct Binding to Ion Channel. Circulation.

[25] Adamiak M., Cheng G., Bobis-Wozowicz S., Zhao L., Kedracka-Krok S., Samanta A., Karnas E., Xuan Y.T., Skupien-Rabian B., Chen X. (2018). Induced Pluripotent Stem Cell (iPSC)-Derived Extracellular Vesicles Are Safer and More Effective for Cardiac Repair Than iPSCs. Circ. Res..

[26] El Harane N., Kervadec A., Bellamy V., Pidial L., Neametalla H.J., Perier M.C., Lima Correa B., Thiébault L., Cagnard N., Duché A. (2018). Acellular therapeutic approach for heart failure: In Vitro production of extracellular vesicles from human cardiovascular progenitors. Eur. Heart J..

[27] Barile L., Lionetti V., Cervio E., Matteucci M., Gherghiceanu M., Popescu L.M., Torre T., Siclari F., Moccetti T., Vassalli G. (2014). Extracellular vesicles from human cardiac progenitor cells inhibit cardiomyocyte apoptosis and improve cardiac function after myocardial infarction. Cardiovasc. Res..

[28] Feng Y., Huang W., Wani M., Yu X., Ashraf M. (2014). Ischemic preconditioning potentiates the protective effect of stem cells through secretion of exosomes by targeting Mecp2 via miR-22. PLoS ONE.

[29] Farahzadi R., Fathi E., Valipour B., Ghaffary S. (2024). Stem cells-derived exosomes as cardiac regenerative agents. IJC Heart Vasc..

[30] Simons M., Raposo G. (2009). Exosomes–vesicular carriers for intercellular communication. Curr. Opin. Cell Biol..

[31] Sundararaj S., Ravindran A., Casarotto M.G. (2021). AHNAK: The quiet giant in calcium homeostasis. Cell Calcium.

[32] Zhang Z., Yao L., Yang J., Wang Z., Du G. (2018). PI3K/Akt and HIF-1 signaling pathway in hypoxia-ischemia (Review). Mol. Med. Rep..

[33] DeBosch B., Sambandam N., Weinheimer C., Courtois M., Muslin A.J. (2006). Akt2 regulates cardiac metabolism and cardiomyocyte survival. J. Biol. Chem..

[34] Taniyama Y., Ito M., Sato K., Kuester C., Veit K., Tremp G., Liao R., Colucci W.S., Ivashchenko Y., Walsh K. (2005). Akt3 overexpression in the heart results in progression from adaptive to maladaptive hypertrophy. J. Mol. Cell. Cardiol..

[35] Sucharski H.C., Dudley E.K., Keith C.B.R., El Refaey M., Koenig S.N., Mohler P.J. (2020). Mechanisms and Alterations of Cardiac Ion Channels Leading to Disease: Role of Ankyrin-B in Cardiac Function. Biomolecules.

[36] Makara M.A., Curran J., Little S.C., Musa H., Polina I., Smith S.A., Wright P.J., Unudurthi S.D., Snyder J., Bennett V. (2014). Ankyrin-G coordinates intercalated disc signaling platform to regulate cardiac excitability in Vivo. Circ. Res..

[37] Dai T., Ramirez-Correa G., Gao W.D. (2006). Apelin increases contractility in failing cardiac muscle. Eur. J. Pharmacol..

[38] Thrasher P., Singh M., Singh K. (2017). Ataxia-Telangiectasia Mutated Kinase: Role in Myocardial Remodeling. J. Rare Dis. Res. Treat..

[39] Barwe S.P., Jordan M.C., Skay A., Inge L., Rajasekaran S.A., Wolle D., Johnson C.L., Neco P., Fang K., Rozengurt N. (2009). Dysfunction of ouabain-induced cardiac contractility in mice with heart-specific ablation of Na,K-ATPase β1-subunit. J. Mol. Cell. Cardiol..

[40] Li J., Wang H., Chen L., Zhong J., Wang J., Xiao J. (2023). Ischemia-reperfusion injury in human AC16 cardiomyocytes is modulated by AXIN1 depending on c-Myc regulation. Ann. Med. Surg..

[41] Su X., Lv L., Li Y., Fang R., Yang R., Li C., Li T., Zhu D., Li X., Zhou Y. (2020). lncRNA MIRF Promotes Cardiac Apoptosis through the miR-26a-Bak1 Axis. Mol. Ther. Nucleic. Acids.

[42] Hochhauser E., Cheporko Y., Yasovich N., Pinchas L., Offen D., Barhum Y., Pannet H., Tobar A., Vidne B.A., Birk E. (2007). Bax deficiency reduces infarct size and improves long-term function after myocardial infarction. Cell Biochem. Biophys..

[43] Mandl A., Huong Pham L., Toth K., Zambetti G., Erhardt P. (2011). Puma deletion delays cardiac dysfunction in murine heart failure models through attenuation of apoptosis. Circulation.

[44] Misao J., Hayakawa Y., Ohno M., Kato S., Fujiwara T., Fujiwara H. (1996). Expression of bcl-2 protein, an inhibitor of apoptosis, and Bax, an accelerator of apoptosis, in ventricular myocytes of human hearts with myocardial infarction. Circulation.

[45] Korshunova A.Y., Blagonravov M.L., Neborak E.V., Syatkin S.P., Sklifasovskaya A.P., Semyatov S.M., Agostinelli E. (2021). BCL2-regulated apoptotic process in myocardial ischemia-reperfusion injury (Review). Int. J. Mol. Med..

[46] Yang W., Han Y., Yang C., Chen Y., Zhao W., Su X., Yang K., Jin W. (2019). MicroRNA-19b-1 reverses ischaemia-induced heart failure by inhibiting cardiomyocyte apoptosis and targeting Bcl2 l11/BIM. Heart Vessel..

[47] Ashkenazi A., Fairbrother W.J., Leverson J.D., Souers A.J. (2017). From basic apoptosis discoveries to advanced selective BCL-2 family inhibitors. Nat. Rev. Drug Discov..

[48] Shukla P.C., Singh K.K., Quan A., Al-Omran M., Teoh H., Lovren F., Cao L., Rovira I.I., Pan Y., Brezden-Masley C. (2011). BRCA1 is an essential regulator of heart function and survival following myocardial infarction. Nat. Commun..

[49] Zhang Q., Chen J., Qin Y., Wang J., Zhou L. (2018). Mutations in voltage-gated L-type calcium channel: Implications in cardiac arrhythmia. Channels.

[50] Wang M.X., Liu X., Li J.M., Liu L., Lu W., Chen G.C. (2020). Inhibition of CACNA1H can alleviate endoplasmic reticulum stress and reduce myocardial cell apoptosis caused by myocardial infarction. Eur. Rev. Med. Pharmacol. Sci..

[51] Ortega A., Tarazón E., Roselló-Lletí E., Gil-Cayuela C., Lago F., González-Juanatey J.R., Cinca J., Jorge E., Martínez-Dolz L., Portolés M. (2015). Patients with Dilated Cardiomyopathy and Sustained Monomorphic Ventricular Tachycardia Show Up-Regulation of KCNN3 and KCNJ2 Genes and CACNG8-Linked Left Ventricular Dysfunction. PLoS ONE.

[52] Yang D., Song L.S., Zhu W.Z., Chakir K., Wang W., Wu C., Wang Y., Xiao R.P., Chen S.R., Cheng H. (2003). Calmodulin regulation of excitation-contraction coupling in cardiac myocytes. Circ. Res..

[53] Condorelli G., Roncarati R., Ross Jr J., Pisani A., Stassi G., Todaro M., Trocha S., Drusco A., Gu Y., Russo M.A. (2001). Heart-targeted overexpression of caspase3 in mice increases infarct size and depresses cardiac function. Proc. Natl. Acad. Sci. USA.

[54] Teringova E., Tousek P. (2017). Apoptosis in ischemic heart disease. J. Transl. Med..

[55] Zhang J., Zhang J., Zhou B., Jiang X., Tang Y., Zhang Z. (2022). Death-Associated Protein Kinase 1 (DAPK1) Protects against Myocardial Injury Induced by Myocardial Infarction in Rats via Inhibition of Inflammation and Oxidative Stress. Dis. Markers.

[56] Kawai T., Nomura F., Hoshino K., Copeland N.G., Gilbert D.J., Jenkins N.A., Akira S. (1999). Death-associated protein kinase 2 is a new calcium/calmodulin-dependent protein kinase that signals apoptosis through its catalytic activity. Oncogene.

[57] Xie L., Pi X., Townley-Tilson W.H., Li N., Wehrens X.H., Entman M.L., Taffet G.E., Mishra A., Peng J., Schisler J.C. (2015). PHD2/3-dependent hydroxylation tunes cardiac response to β-adrenergic stress via phospholamban. J. Clin. Investig..

[58] Xin Z., Ma Z., Jiang S., Wang D., Fan C., Di S., Hu W., Li T., She J., Yang Y. (2017). FOXOs in the impaired heart: New therapeutic targets for cardiac diseases. Biochim. Et Biophys. Acta (BBA) Mol. Basis Dis..

[59] Sato P.Y., Chuprun J.K., Ibetti J., Cannavo A., Drosatos K., Elrod J.W., Koch W.J. (2015). GRK2 compromises cardiomyocyte mitochondrial function by diminishing fatty acid-mediated oxygen consumption and increasing superoxide levels. J. Mol. Cell. Cardiol..

[60] Datta Chaudhuri R., Banik A., Mandal B., Sarkar S. (2021). Cardiac-specific overexpression of HIF-1α during acute myocardial infarction ameliorates cardiomyocyte apoptosis via differential regulation of hypoxia-inducible pro-apoptotic and anti-oxidative genes. Biochem. Biophys. Res. Commun..

[61] Yeghiazarians Y., Honbo N., Imhof I., Woods B., Aguilera V., Ye J., Boyle A.J., Karliner J.S. (2014). IL-15: A novel prosurvival signaling pathway in cardiomyocytes. J. Cardiovasc. Pharmacol..

[62] Pineda S., Nikolova-Krstevski V., Leimena C., Atkinson A.J., Altekoester A.K., Cox C.D., Jacoby A., Huttner I.G., Ju Y.K., Soka M. (2021). Conserved Role of the Large Conductance Calcium-Activated Potassium Channel, K(Ca)1.1, in Sinus Node Function and Arrhythmia Risk. Circ. Genom. Precis. Med..

[63] Rasmussen M.L., Taneja N., Neininger A.C., Wang L., Robertson G.L., Riffle S.N., Shi L., Knollmann B.C., Burnette D.T., Gama V. (2020). MCL-1 Inhibition by Selective BH3 Mimetics Disrupts Mitochondrial Dynamics Causing Loss of Viability and Functionality of Human Cardiomyocytes. iScience.

[64] Sciarretta S., Volpe M., Sadoshima J. (2014). Mammalian target of rapamycin signaling in cardiac physiology and disease. Circ. Res..

[65] Movsesian M., Ahmad F., Hirsch E. (2018). Functions of PDE3 Isoforms in Cardiac Muscle. J. Cardiovasc. Dev. Dis..

[66] Mora A., Davies A.M., Bertrand L., Sharif I., Budas G.R., Jovanović S., Mouton V., Kahn C.R., Lucocq J.M., Gray G.A. (2003). Deficiency of PDK1 in cardiac muscle results in heart failure and increased sensitivity to hypoxia. Embo J..

[67] Nicolaou P., Kranias E.G. (2009). Role of PP1 in the regulation of Ca cycling in cardiac physiology and pathophysiology. Front. Biosci. (Landmark Ed.).

[68] Liu Q., Chen X., Macdonnell S.M., Kranias E.G., Lorenz J.N., Leitges M., Houser S.R., Molkentin J.D. (2009). Protein kinase C[alpha], but not PKC[beta] or PKC[gamma], regulates contractility and heart failure susceptibility: Implications for ruboxistaurin as a novel therapeutic approach. Circ. Res..

[69] McCarthy J., Lochner A., Opie L.H., Sack M.N., Essop M.F. (2011). PKCε promotes cardiac mitochondrial and metabolic adaptation to chronic hypobaric hypoxia by GSK3β inhibition. J. Cell. Physiol..

[70] Carpi A., Menabò R., Kaludercic N., Pelicci P., Di Lisa F., Giorgio M. (2009). The cardioprotective effects elicited by p66(Shc) ablation demonstrate the crucial role of mitochondrial ROS formation in ischemia/reperfusion injury. Biochim. Et Biophys. Acta (BBA)-Bioenerg..

[71] Kokoszka J.E., Waymire K.G., Flierl A., Sweeney K.M., Angelin A., MacGregor G.R., Wallace D.C. (2016). Deficiency in the mouse mitochondrial adenine nucleotide translocator isoform 2 gene is associated with cardiac noncompaction. Biochim. Et Biophys. Acta: Int. J. Biochem. Biophys..

[72] Zhang L., Lv L., Zheng N., Li R., Yang R., Li T., Li Y., Liu Y., Luo H., Li X. (2021). Suppression of Sox4 protects against myocardial ischemic injury by reduction of cardiac apoptosis in mice. J. Cell. Physiol..

[73] Piacentino III V., Milano C.A., Bolanos M., Schroder J., Messina E., Cockrell A.S., Jones E., Krol A., Bursac N., Mao L. (2012). X-linked inhibitor of apoptosis protein-mediated attenuation of apoptosis, using a novel cardiac-enhanced adeno-associated viral vector. Hum. Gene. Ther..

[74] DiFrancesco D. (2015). HCN4, Sinus Bradycardia and Atrial Fibrillation. Arrhythm. Electrophysiol. Rev..

[75] Ferrari R., Fox K. (2016). Heart rate reduction in coronary artery disease and heart failure. Nat. Rev. Cardiol..

[76] Bers D. (2001). Excitation-Contraction Coupling and Cardiac Contractile Force.

[77] Sun Y.B., Irving M. (2010). The molecular basis of the steep force-calcium relation in heart muscle. J. Mol. Cell. Cardiol..

[78] Pandey V., Xie L.H., Qu Z., Song Z. (2021). Mitochondrial depolarization promotes calcium alternans: Mechanistic insights from a ventricular myocyte model. PLoS Comput. Biol..

[79] Florea S.M., Blatter L.A. (2010). The role of mitochondria for the regulation of cardiac alternans. Front. Physiol..

[80] Kohlhaas M., Nickel A.G., Maack C. (2017). Mitochondrial energetics and calcium coupling in the heart. J. Physiol..

[81] Sun B., Zhao C., Mao Y. (2021). MiR-218-5p Mediates Myocardial Fibrosis after Myocardial Infarction by Targeting CX43. Curr. Pharm. Des..

[82] Zhang J., Zhou X., Sun J., Li M., Ma J., Ge L. (2022). miR-361-3p mitigates hypoxia-induced cardiomyocyte injury via targeting apoptosis initiators caspase-2/-8/-9. Vitr. Cell Dev. Biol. Anim..

[83] Zhang C., Zhang H., Zhao L., Wei Z., Lai Y., Ma X. (2022). Differential Expression of microRNAs in Hypertrophied Myocardium and Their Relationship to Late Gadolinium Enhancement, Left Ventricular Hypertrophy and Remodeling in Hypertrophic Cardiomyopathy. Diagnostics.

[84] Yan K., An T., Zhai M., Huang Y., Wang Q., Wang Y., Zhang R., Wang T., Liu J., Zhang Y. (2019). Mitochondrial miR-762 regulates apoptosis and myocardial infarction by impairing ND2. Cell Death Dis..

[85] Mathivanan S., Ji H., Simpson R.J. (2010). Exosomes: Extracellular organelles important in intercellular communication. J. Proteom..

[86] Marwarha G., Røsand Ø., Scrimgeour N., Slagsvold K.H., Høydal M.A. (2021). miR-210 regulates apoptotic cell death during cellular hypoxia and reoxygenation in a diametrically opposite manner. Biomedicines.

[87] Marwarha G., Røsand Ø., Slagsvold K.H., Høydal M.A. (2022). GSK3β Inhibition Is the Molecular Pivot That Underlies the Mir-210-Induced Attenuation of Intrinsic Apoptosis Cascade during Hypoxia. Int. J. Mol. Sci..

[88] Hayes H.B., Nicolini A.M., Arrowood C.A., Chvatal S.A., Wolfson D.W., Cho H.C., Sullivan D.D., Chal J., Fermini B., Clements M. (2019). Novel method for action potential measurements from intact cardiac monolayers with multiwell microelectrode array technology. Sci. Rep..

[89] Love M.I., Huber W., Anders S. (2014). Moderated estimation of fold change and dispersion for RNA-seq data with DESeq2. Genome Biol..

[90] Tastsoglou S., Skoufos G., Miliotis M., Karagkouni D., Koutsoukos I., Karavangeli A., Kardaras F.S., Hatzigeorgiou A.G. (2023). DIANA-miRPath v4. 0: Expanding target-based miRNA functional analysis in cell-type and tissue contexts. Nucleic Acids Res..

[91] Xiao F., Zuo Z., Cai G., Kang S., Gao X., Li T. (2009). miRecords: An integrated resource for microRNA–target interactions. Nucleic Acids Res..

[92] Huang H.-Y., Lin Y.-C.-D., Cui S., Huang Y., Tang Y., Xu J., Bao J., Li Y., Wen J., Zuo H. (2022). miRTarBase update 2022: An informative resource for experimentally validated miRNA–target interactions. Nucleic Acids Res..

[93] Karagkouni D., Paraskevopoulou M.D., Chatzopoulos S., Vlachos I.S., Tastsoglou S., Kanellos I., Papadimitriou D., Kavakiotis I., Maniou S., Skoufos G. (2018). DIANA-TarBase v8: A decade-long collection of experimentally supported miRNA–gene interactions. Nucleic Acids Res..

[94] Ru Y., Kechris K.J., Tabakoff B., Hoffman P., Radcliffe R.A., Bowler R., Mahaffey S., Rossi S., Calin G.A., Bemis L. (2014). The multiMiR R package and database: Integration of microRNA–target interactions along with their disease and drug associations. Nucleic Acids Res..

[95] Wu T., Hu E., Xu S., Chen M., Guo P., Dai Z., Feng T., Zhou L., Tang W., Zhan L. (2021). Clusterprofiler 4.0: A universal enrichment tool for interpreting omics data. Innov. Camb..

[96] Thomas P.D., Ebert D., Muruganujan A., Mushayahama T., Albou L.P., Mi H. (2022). PANTHER: Making genome—Scale phylogenetics accessible to all. Protein Sci..

[97] Kanehisa M., Goto S. (2000). KEGG: Kyoto encyclopedia of genes and genomes. Nucleic Acids Res..

[98] R Core Team R. (2013). R: A Language and Environment for Statistical Computing.

[99] Wickham H., Averick M., Bryan J., Chang W., McGowan L.D., François R., Grolemund G., Hayes A., Henry L., Hester J. (2019). Welcome to the tidyverse. J. Open Source Softw..

[100] Carlson M., Pages H., Li N. (2019). Carlson M (2019). org.Hs.eg.db: Genome Wide Annotation for Human. R Package.

[101] Yu G., Wang L.-G., Yan G.-R., He Q.-Y. (2014). DOSE: An R/Bioconductor package for disease ontology semantic and enrichment analysis. Bioinformatics.

[102] Ambrosy A.P., Fonarow G.C., Butler J., Chioncel O., Greene S.J., Vaduganathan M., Nodari S., Lam C.S.P., Sato N., Shah A.N. (2014). The global health and economic burden of hospitalizations for heart failure: Lessons learned from hospitalized heart failure registries. J. Am. Coll. Cardiol..

