# Supplementary material for: Exosomal Preconditioning of Human iPSC-Derived Cardiomyocytes Beneficially Alters Cardiac Electrophysiology and Micro RNA Expression
Source: Int J Mol Sci. 2024 Aug 2;25(15):8460. doi: 10.3390/ijms25158460 (PMC11313350; doi:10.3390/ijms25158460)
Supplement: Supplementary file 1 [file ijms-25-08460-s001.zip › ijms-3081002-supplementary.pdf]

Table S1

Component	Amount 20mL	Final concentration		50mL total
DMEM, no Glucose, no Glutamine, no Phenol Red	18.578 mL	93%		46.45 mL
Creatine (hydrous)	13.12 mg	5 mM		32.8 mg
D-(+) Glucose Solution 2.5 M, 450 g/L	0.022 mL	2,75 mM		55uL
Glutamine 200 mM	0.2 mL	2 mM		500uL
HEPES 1M	0.2 mL	10 mM		500uL
L-carnitine 200 mM	0.2 mL	2 mM		500uL
Non-essential Amino Acids 100X	0.2 mL	1X		500uL
Sodium Pyruvate 100 mM	0.2 mL	1 mM		500uL
Taurine 500 mM	0.2 mL	5 mM		500uL
Linoleic-oleic Acid 100X (add after filtering the rest)	0.2 mL	1X		500uL

Supplementary Table S1: Notation legend

1. Thermo Fisher Scientific, Oslo, Norway, Catalogue # 11966025

2. Sigma Aldrich / Merck Millipore / Merck Life Science, Darmstadt, Germany, Catalogue # C3630-100G

3. Sigma Aldrich / Merck Millipore / Merck Life Science, Oslo, Norway, Catalogue # G8769

4. Thermo Fisher Scientific, Oslo, Norway, Catalogue # A2916801

5. Sigma Aldrich / Merck Millipore / Merck Life Science, Darmstadt, Germany, Catalogue # H4034-500G

6. Sigma Aldrich / Merck Millipore / Merck Life Science, Darmstadt, Germany, Catalogue # C0283-25G

7. Thermo Fisher Scientific, Oslo, Norway, Catalogue # 11140035

8. Thermo Fisher Scientific, Oslo, Norway, Catalogue # 11360070

9. Sigma Aldrich / Merck Millipore / Merck Life Science, Darmstadt, Germany, Catalogue # T8691-100G

10. Sigma Aldrich / Merck Millipore / Merck Life Science, Darmstadt, Germany, Catalogue # L9655-5ML

* N/A - Not Applicable

	baseMean	log2FoldChange	lfcSE	stat	pvalue	padj
let-7a-1-5p	23,218	-0,004	0,283	-0,014	0,989	0,995
let-7a-2-3p	70,251	-0,340	0,310	-1,099	0,272	0,967
let-7a-2-5p	4875,529	0,222	0,158	1,401	0,161	0,894
let-7a-3p	32,150	0,106	0,305	0,348	0,728	0,995
let-7a-5p	32884,362	0,118	0,147	0,801	0,423	0,995
let-7b-3p	7,409	-0,030	0,191	-0,158	0,875	0,995
let-7b-5p	421,865	0,035	0,231	0,152	0,880	0,995
let-7c-3p	4,146	0,008	0,121	0,068	NA	NA
let-7c-5p	1232,740	0,173	0,181	0,955	0,340	0,995
let-7d-3p	346,542	-0,580	0,297	-1,951	0,051	0,845
let-7d-5p	4284,383	0,226	0,161	1,404	0,160	0,894
let-7e-3p	51,416	0,391	0,316	1,241	0,215	0,930
let-7e-5p	4450,915	0,237	0,210	1,125	0,260	0,954
let-7f-1-3p	39,733	0,321	0,316	1,016	0,310	0,987
let-7f-1-5p	2680,373	0,020	0,155	0,130	0,896	0,995
let-7f-2-3p	8,746	-0,027	0,163	-0,164	0,870	0,995
let-7f-2-5p	530,922	0,182	0,203	0,894	0,371	0,995
let-7f-5p	34171,390	0,097	0,134	0,729	0,466	0,995
let-7g-5p	7908,096	0,154	0,201	0,769	0,442	0,995
let-7i-5p	6249,474	0,251	0,154	1,629	0,103	0,892
miR-100-3p	59,117	-0,376	0,316	-1,193	0,233	0,948
miR-100-5p	141796,488	0,039	0,164	0,238	0,812	0,995
miR-101-2-3p	14,374	0,103	0,174	0,622	0,534	0,995
miR-10394-3p	2,262	0,049	0,104	0,540	0,589	0,995
miR-10395-3p	6,064	-0,104	0,200	-0,532	0,595	0,995
miR-10398-3p	6,061	-0,167	0,178	-1,078	0,281	0,970
miR-103a-1-3p	1736,586	0,156	0,182	0,859	0,391	0,995
miR-103a-2-3p	52,927	0,352	0,316	1,114	0,265	0,954
miR-103a-2-5p	3,957	0,051	0,120	0,456	0,648	0,995
miR-103a-3p	5636,486	0,186	0,168	1,106	0,269	0,961
miR-10400-3p	2,625	-0,088	0,107	-1,936	0,053	0,845
miR-10400-5p	81,289	0,007	0,310	0,023	0,982	0,995
miR-10401-3p	5,210	0,151	0,140	2,028	0,043	0,845

miR-10522-5p	0,420	-0,080	0,103	-1,205	0,228	0,948
miR-10523-5p	7,568	-0,096	0,150	-0,704	0,481	0,995
miR-10524-5p	2,874	0,047	0,104	0,510	NA	NA
miR-106a-5p	3,420	0,116	0,143	0,993	NA	NA
miR-106b-3p	71,980	-0,164	0,308	-0,532	0,595	0,995
miR-106b-5p	331,718	-0,306	0,220	-1,391	0,164	0,894
miR-107	341,620	0,058	0,267	0,218	0,828	0,995
miR-107-3p	528,180	0,237	0,221	1,072	0,284	0,970
miR-10a-5p	9564,021	0,037	0,180	0,205	0,838	0,995
miR-10b-5p	124,035	0,734	0,307	2,489	0,013	0,470
miR-1-1-3p	7,596	0,037	0,234	0,158	0,874	0,995
miR-1179-5p	3,503	-0,105	0,117	-2,220	0,026	0,747
miR-1180-3p	294,907	0,107	0,256	0,420	0,675	0,995
miR-1181-3p	3,135	-0,098	0,113	-2,105	0,035	0,829
miR-1181-5p	0,000	NA	NA	NA	NA	NA
miR-1183-3p	8,079	0,026	0,157	0,167	0,867	0,995
miR-1199-5p	4,682	0,044	0,151	0,295	0,768	0,995
miR-1202-3p	4,625	-0,176	0,147	-2,913	0,004	0,306
miR-12114-5p	1,145	0,077	0,104	1,345	0,179	0,894
miR-12116-3p	2,099	-0,077	0,104	-1,295	0,195	0,896
miR-12121-3p	4,489	0,016	0,122	0,131	0,895	0,995
miR-12124-3p	7,638	0,060	0,135	0,468	0,640	0,995
miR-12126-5p	37,358	0,166	0,239	0,711	0,477	0,995
miR-12131-5p	4,968	0,086	0,106	2,184	NA	NA
miR-12135-3p	743,123	-0,524	0,274	-1,910	0,056	0,851
miR-12136	307,727	0,551	0,257	2,144	0,032	0,814
miR-12136-3p	301,959	0,032	0,249	0,129	0,897	0,995
miR-12136-5p	608,195	0,002	0,227	0,009	0,993	0,995
miR-1224-5p	20,451	0,255	0,233	1,170	0,242	0,954
miR-122-5p	104,600	0,282	0,316	0,893	0,372	0,995
miR-1226-3p	328,774	-0,139	0,291	-0,477	0,634	0,995
miR-1227-3p	1,898	0,083	0,104	1,789	0,074	0,853
miR-1227-5p	1,359	0,082	0,104	1,665	0,096	0,892
miR-1228-3p	0,634	0,046	0,103	0,499	0,618	0,995

miR-1229-3p	3,246	0,012	0,115	0,108	0,914	0,995
miR-122b-5p	23,304	0,183	0,298	0,616	0,538	0,995
miR-1233-2-5p	175,905	0,607	0,304	2,000	0,046	0,845
miR-1234-5p	580,376	1,277	0,241	5,305	0,000	0,000
miR-1237-3p	0,757	0,081	0,103	1,447	0,148	0,894
miR-1238-5p	0,052	0,002	0,096	0,023	0,982	0,995
miR-1-2-3p	439,985	0,072	0,291	0,249	0,804	0,995
miR-1246	10,285	-0,094	0,208	-0,460	0,646	0,995
miR-1246-5p	46,935	0,300	0,316	0,951	0,342	0,995
miR-1247-3p	2,923	0,037	0,104	0,384	NA	NA
miR-1247-5p	0,604	-0,081	0,103	-1,340	0,180	0,894
miR-1248-3p	1,811	-0,109	0,145	-0,880	0,379	0,995
miR-1248-5p	4,767	0,129	0,128	2,596	0,009	0,470
miR-1249-3p	21,627	0,285	0,284	1,019	0,308	0,987
miR-1249-5p	8,307	0,095	0,194	0,503	0,615	0,995
miR-1256-3p	8,064	-0,138	0,167	-0,937	0,349	0,995
miR-125a-3p	135,204	0,260	0,303	0,858	0,391	0,995
miR-125a-5p	16247,573	-0,152	0,215	-0,707	0,480	0,995
miR-125b-1-3p	173,963	0,168	0,282	0,596	0,551	0,995
miR-125b-1-5p	77,087	-0,369	0,316	-1,174	0,241	0,954
miR-125b-2-3p	77,427	-0,252	0,316	-0,798	0,425	0,995
miR-125b-2-5p	7045,774	-0,400	0,315	-1,277	0,202	0,903
miR-125b-5p	19041,892	0,090	0,261	0,343	0,732	0,995
miR-1260a-5p	779,802	0,052	0,275	0,191	0,849	0,995
miR-1260b-5p	76,916	0,062	0,316	0,198	0,843	0,995
miR-1261-5p	85,801	0,122	0,309	0,396	0,692	0,995
miR-1262-5p	4,339	0,091	0,126	0,909	0,363	0,995
miR-126-3p	5676,458	-0,018	0,222	-0,079	0,937	0,995
miR-126-5p	1198,231	0,017	0,229	0,074	0,941	0,995
miR-1267-3p	3,471	0,071	0,140	0,543	0,587	0,995
miR-1268a-3p	1,335	0,078	0,104	1,404	0,160	0,894
miR-1268a-5p	18,704	-0,239	0,221	-1,170	0,242	0,954
miR-1268b-3p	10,160	-0,016	0,249	-0,066	0,947	0,995
miR-1268b-5p	0,757	0,081	0,103	1,447	0,148	0,894

miR-1269a	0,543	0,081	0,103	1,324	0,185	0,894
miR-1269a-3p	0,263	-0,078	0,101	-1,029	0,303	0,987
miR-1271-5p	55,421	0,104	0,315	0,330	0,742	0,995
miR-1273c-3p	18,574	-0,341	0,302	-1,144	0,253	0,954
miR-1273c-5p	1,308	0,078	0,104	1,396	0,163	0,894
miR-1273h-5p	2,121	-0,029	0,104	-0,293	0,770	0,995
miR-127-3p	421,693	-0,044	0,288	-0,153	0,879	0,995
miR-1275-5p	32,957	0,426	0,316	1,354	0,176	0,894
miR-1276-5p	5,334	-0,139	0,185	-0,806	0,420	0,995
miR-1281-3p	3,970	0,115	0,121	2,386	0,017	0,552
miR-128-1-3p	305,404	-0,243	0,287	-0,847	0,397	0,995
miR-128-1-5p	7,497	0,282	0,186	2,538	0,011	0,470
miR-128-2-3p	26,057	0,055	0,299	0,184	0,854	0,995
miR-128-3p	1546,366	-0,276	0,237	-1,166	0,244	0,954
miR-1284-5p	1,145	-0,015	0,104	-0,150	0,881	0,995
miR-1285-1-3p	13,523	-0,012	0,206	-0,058	0,953	0,995
miR-1285-1-5p	3,850	0,032	0,118	0,280	0,779	0,995
miR-1285-2-5p	8,208	0,079	0,162	0,506	0,613	0,995
miR-1287-5p	167,401	0,525	0,265	1,984	0,047	0,845
miR-1289-1-3p	0,400	-0,066	0,102	-0,910	0,363	0,995
miR-1290-3p	34,415	-0,230	0,313	-0,735	0,462	0,995
miR-1291-3p	0,000	NA	NA	NA	NA	NA
miR-1291-5p	11,323	0,124	0,241	0,522	0,602	0,995
miR-1292-5p	7,334	0,067	0,194	0,351	0,726	0,995
miR-1293-5p	5,117	0,264	0,175	3,523	0,000	0,067
miR-1296-3p	1,443	0,019	0,104	0,185	0,853	0,995
miR-1296-5p	10,934	0,031	0,202	0,156	0,876	0,995
miR-1301-3p	2157,337	0,051	0,209	0,242	0,809	0,995
miR-1303	6,185	0,054	0,154	0,360	0,719	0,995
miR-1303-3p	27,928	0,166	0,307	0,543	0,587	0,995
miR-1303-5p	19,389	-0,289	0,234	-1,343	0,179	0,894
miR-1304-3p	69,652	0,498	0,312	1,598	0,110	0,892
miR-1304-5p	16,976	0,043	0,217	0,199	0,842	0,995
miR-1306-3p	10,467	-0,167	0,213	-0,820	0,412	0,995

miR-1306-5p	72,088	0,091	0,296	0,309	0,757	0,995
miR-1307-3p	154,819	0,418	0,277	1,506	0,132	0,894
miR-1307-5p	1,877	0,017	0,104	0,169	0,866	0,995
miR-130a-3p	2831,644	-0,060	0,188	-0,322	0,747	0,995
miR-130b-3p	196,671	0,446	0,284	1,575	0,115	0,894
miR-130b-5p	79,114	0,464	0,312	1,489	0,136	0,894
miR-132-3p	30,425	0,009	0,305	0,030	0,976	0,995
miR-132-5p	102,349	0,046	0,315	0,146	0,884	0,995
miR-133a-1-3p	1474,978	0,232	0,216	1,074	0,283	0,970
miR-133a-1-5p	14,134	-0,057	0,269	-0,210	0,833	0,995
miR-133a-3p	68,566	0,508	0,314	1,624	0,104	0,892
miR-133a-5p	28,157	-0,258	0,290	-0,900	0,368	0,995
miR-133b-3p	3,617	-0,026	0,155	-0,172	0,864	0,995
miR-1343-3p	6,874	0,139	0,163	0,988	0,323	0,995
miR-1343-5p	10,585	-0,056	0,231	-0,245	0,807	0,995
miR-135a-2-3p	0,000	NA	NA	NA	NA	NA
miR-135b-5p	6,193	0,002	0,131	0,015	NA	NA
miR-137-3p	157,300	-0,272	0,303	-0,900	0,368	0,995
miR-138-1-5p	50,593	0,249	0,311	0,803	0,422	0,995
miR-138-2-3p	4,793	-0,022	0,138	-0,160	0,873	0,995
miR-138-2-5p	27,170	0,311	0,299	1,052	0,293	0,983
miR-138-5p	76,254	-0,107	0,308	-0,347	0,729	0,995
miR-139-3p	8,952	-0,120	0,247	-0,489	0,625	0,995
miR-139-5p	676,264	0,231	0,221	1,048	0,295	0,986
miR-1-3p	2880,532	0,051	0,316	0,162	0,872	0,995
miR-140-3p	132,278	-0,006	0,283	-0,020	0,984	0,995
miR-140-5p	21,576	0,044	0,104	0,467	0,640	0,995
miR-141-3p	7,953	0,075	0,191	0,399	0,690	0,995
miR-142-5p	4,050	0,008	0,134	0,060	0,952	0,995
miR-143-3p	126859,321	-0,012	0,168	-0,070	0,944	0,995
miR-143-5p	7,120	-0,030	0,135	-0,223	NA	NA
miR-145-3p	1285,673	-0,136	0,240	-0,566	0,572	0,995
miR-145-5p	16025,387	0,062	0,151	0,409	0,682	0,995
miR-1468-5p	11,448	-0,080	0,208	-0,387	0,699	0,995

miR-1469-5p	49,362	0,248	0,290	0,864	0,387	0,995
miR-146a-5p	7980,274	-0,217	0,217	-1,000	0,317	0,995
miR-146b-5p	515,103	0,259	0,212	1,224	0,221	0,947
miR-148a-3p	959,246	-0,393	0,245	-1,601	0,109	0,892
miR-148b-3p	525,281	-0,043	0,248	-0,174	0,862	0,995
miR-148b-5p	6,889	0,120	0,138	1,151	0,250	0,954
miR-149-5p	4212,767	0,122	0,168	0,725	0,469	0,995
miR-150-5p	0,973	0,082	0,104	1,540	0,123	0,894
miR-151a-3p	3703,953	0,097	0,188	0,516	0,606	0,995
miR-151a-5p	8948,848	0,083	0,153	0,546	0,585	0,995
miR-151b	19,743	0,341	0,291	1,189	0,234	0,948
miR-151b-3p	163,231	0,481	0,268	1,796	0,072	0,853
miR-152-3p	1381,850	-0,166	0,188	-0,884	0,377	0,995
miR-153-2-5p	4,049	0,083	0,104	2,069	NA	NA
miR-155-5p	27,815	0,102	0,289	0,354	0,724	0,995
miR-15a-5p	24,023	0,066	0,299	0,220	0,826	0,995
miR-15b-3p	7,954	-0,271	0,226	-1,313	0,189	0,894
miR-15b-5p	8819,354	-0,101	0,227	-0,444	0,657	0,995
miR-16-1-5p	2988,257	0,096	0,141	0,683	0,494	0,995
miR-16-2-3p	6,338	-0,015	0,184	-0,082	0,934	0,995
miR-16-2-5p	29,933	-0,076	0,310	-0,244	0,808	0,995
miR-16-5p	17914,839	0,055	0,129	0,423	0,673	0,995
miR-17-3p	42,320	0,071	0,242	0,295	0,768	0,995
miR-17-5p	862,236	0,022	0,198	0,109	0,913	0,995
miR-181a-1-3p	117,878	-0,047	0,310	-0,151	0,880	0,995
miR-181a-1-5p	1,985	0,032	0,104	0,318	0,750	0,995
miR-181a-2-3p	7462,442	0,057	0,156	0,366	0,715	0,995
miR-181a-2-5p	4738,497	0,445	0,178	2,504	0,012	0,470
miR-181a-3p	2424,541	-0,073	0,187	-0,388	0,698	0,995
miR-181a-5p	2587,537	0,174	0,184	0,946	0,344	0,995
miR-181b-1-5p	246,584	-0,010	0,273	-0,036	0,972	0,995
miR-181b-2-3p	13,842	0,156	0,213	0,762	0,446	0,995
miR-181b-2-5p	106,408	-0,005	0,301	-0,017	0,986	0,995
miR-181b-5p	53,208	0,610	0,314	1,972	0,049	0,845

miR-181c-3p	396,582	0,124	0,208	0,598	0,550	0,995
miR-181c-5p	15,679	0,109	0,218	0,507	0,612	0,995
miR-181d-3p	0,989	0,004	0,104	0,034	0,973	0,995
miR-181d-5p	31,756	-0,182	0,285	-0,643	0,520	0,995
miR-182-5p	81,241	0,300	0,303	0,991	0,322	0,995
miR-1827-3p	12,638	0,101	0,255	0,400	0,689	0,995
miR-183-5p	44,500	0,282	0,315	0,898	0,369	0,995
miR-1843-3p	52,516	-0,359	0,316	-1,139	0,255	0,954
miR-1843-5p	52,979	0,038	0,312	0,121	0,903	0,995
miR-184-3p	7,211	-0,161	0,194	-0,896	0,370	0,995
miR-185-5p	818,449	0,123	0,219	0,562	0,574	0,995
miR-186-5p	189,823	-0,201	0,291	-0,691	0,490	0,995
miR-187-3p	14,645	0,119	0,216	0,561	0,575	0,995
miR-188-3p	2,287	-0,083	0,104	-1,834	0,067	0,853
miR-18a-3p	4,579	-0,146	0,176	-0,924	0,355	0,995
miR-18a-5p	213,135	-0,358	0,263	-1,361	0,174	0,894
miR-18b-5p	0,508	-0,070	0,103	-1,005	0,315	0,995
miR-1908-3p	6,366	0,031	0,131	0,239	0,811	0,995
miR-1908-5p	198,642	0,082	0,261	0,313	0,754	0,995
miR-1909-5p	1,107	-0,073	0,104	-1,072	0,284	0,970
miR-190a-3p	0,368	-0,079	0,102	-1,155	0,248	0,954
miR-190a-5p	1,566	-0,030	0,104	-0,304	0,761	0,995
miR-1910-3p	9,439	-0,005	0,162	-0,032	0,974	0,995
miR-1911-3p	6,311	0,009	0,155	0,056	0,956	0,995
miR-191-3p	136,454	-0,331	0,296	-1,120	0,263	0,954
miR-1915-3p	2,606	0,056	0,125	0,486	0,627	0,995
miR-1915-5p	0,529	0,043	0,103	0,462	0,644	0,995
miR-191-5p	47466,743	0,081	0,175	0,460	0,646	0,995
miR-192-5p	63,045	-0,026	0,279	-0,094	0,925	0,995
miR-193a-3p	3,536	0,087	0,121	0,933	0,351	0,995
miR-193a-5p	79,644	-0,077	0,315	-0,245	0,806	0,995
miR-193b-3p	165,595	0,082	0,277	0,296	0,767	0,995
miR-193b-5p	80,175	0,412	0,314	1,320	0,187	0,894
miR-194-1-5p	3,470	0,286	0,199	1,869	0,062	0,853

miR-194-2-5p	61,737	0,034	0,308	0,111	0,912	0,995
miR-194-5p	58,472	-0,060	0,316	-0,190	0,849	0,995
miR-195-5p	57,448	0,094	0,313	0,300	0,764	0,995
miR-196a-1-5p	2,874	0,119	0,125	2,011	0,044	0,845
miR-1973-3p	5,274	-0,141	0,158	-1,063	0,288	0,974
miR-197-3p	359,731	0,101	0,270	0,376	0,707	0,995
miR-197-5p	0,000	NA	NA	NA	NA	NA
miR-1976-5p	15,106	0,081	0,179	0,466	0,641	0,995
miR-199a-2-3p	3277,702	0,219	0,232	0,944	0,345	0,995
miR-199a-2-5p	32,971	0,057	0,293	0,194	0,846	0,995
miR-199a-5p	70,354	0,065	0,316	0,204	0,838	0,995
miR-199b-3p	1799,154	0,137	0,261	0,526	0,599	0,995
miR-199b-5p	9,559	-0,028	0,254	-0,110	0,912	0,995
miR-19a-3p	16,951	-0,025	0,220	-0,114	0,909	0,995
miR-19b-2-3p	1,584	-0,120	0,131	-1,388	0,165	0,894
miR-19b-3p	6,669	-0,056	0,188	-0,303	0,762	0,995
miR-200b-3p	5,440	-0,206	0,160	-2,566	0,010	0,470
miR-200b-5p	2,736	-0,039	0,104	-0,410	0,682	0,995
miR-200c-3p	54,038	0,415	0,315	1,322	0,186	0,894
miR-202-3p	1,489	-0,010	0,104	-0,100	0,920	0,995
miR-202-5p	1,005	0,082	0,104	1,553	0,120	0,894
miR-203a-3p	44,028	-0,281	0,311	-0,905	0,366	0,995
miR-203a-5p	5,840	-0,066	0,131	-0,553	0,581	0,995
miR-204-3p	0,540	0,081	0,103	1,322	0,186	0,894
miR-204-5p	1,908	-0,012	0,104	-0,112	0,911	0,995
miR-208a-3p	1126,621	-0,314	0,255	-1,231	0,218	0,941
miR-208a-5p	2794,320	0,028	0,229	0,122	0,903	0,995
miR-208b-3p	4846,075	-0,340	0,253	-1,346	0,178	0,894
miR-208b-5p	0,683	-0,081	0,103	-1,385	0,166	0,894
miR-20a-3p	5,929	-0,039	0,143	-0,279	0,780	0,995
miR-20a-5p	187,630	0,302	0,271	1,113	0,266	0,954
miR-20b-5p	2,214	-0,129	0,130	-1,940	0,052	0,845
miR-210-3p	1375,066	0,246	0,218	1,128	0,259	0,954
miR-210-5p	5,032	0,021	0,126	0,168	0,867	0,995

miR-2110	18,416	0,094	0,220	0,430	0,667	0,995
miR-2110-3p	5,258	-0,093	0,130	-0,870	0,384	0,995
miR-2110-5p	320,309	0,148	0,253	0,585	0,559	0,995
miR-2113-3p	3,508	-0,013	0,116	-0,116	0,908	0,995
miR-2116-3p	1,419	0,030	0,104	0,299	0,765	0,995
miR-2117-3p	7,063	-0,017	0,210	-0,082	0,935	0,995
miR-212-3p	5,676	0,134	0,184	0,775	0,439	0,995
miR-212-5p	3,714	0,099	0,124	1,118	0,263	0,954
miR-21-3p	60,145	-0,023	0,316	-0,072	0,943	0,995
miR-214-3p	1232,510	-0,129	0,271	-0,478	0,633	0,995
miR-214-5p	7,783	-0,100	0,192	-0,536	0,592	0,995
miR-21-5p	25187,402	-0,347	0,197	-1,759	0,079	0,853
miR-216a-3p	6,670	0,006	0,157	0,039	0,969	0,995
miR-216a-5p	2,017	0,032	0,104	0,327	0,743	0,995
miR-218-1-3p	19,038	-0,014	0,300	-0,048	0,962	0,995
miR-218-1-5p	0,342	-0,079	0,102	-1,127	0,260	0,954
miR-218-2-3p	3,409	0,023	0,111	0,214	NA	NA
miR-218-2-5p	119,591	-0,064	0,298	-0,216	0,829	0,995
miR-218-5p	40,342	-0,985	0,316	-3,209	0,001	0,179
miR-219a-1-3p	11,095	0,089	0,190	0,479	0,632	0,995
miR-219a-1-5p	0,919	0,082	0,104	1,519	0,129	0,894
miR-219b-5p	1,631	0,021	0,104	0,204	0,838	0,995
miR-221-3p	2531,585	0,003	0,168	0,021	0,984	0,995
miR-221-5p	1344,801	-0,199	0,166	-1,197	0,231	0,948
miR-222-3p	997,429	-0,387	0,230	-1,687	0,092	0,892
miR-222-5p	78,881	-0,188	0,305	-0,617	0,537	0,995
miR-223-3p	1,273	0,082	0,104	1,641	0,101	0,892
miR-22-3p	6129,152	-0,195	0,203	-0,960	0,337	0,995
miR-224-3p	66,018	-0,006	0,307	-0,020	0,984	0,995
miR-224-5p	1388,072	0,135	0,197	0,687	0,492	0,995
miR-22-5p	994,811	-0,153	0,258	-0,592	0,554	0,995
miR-2276-3p	0,883	0,009	0,104	0,083	0,934	0,995
miR-2277-5p	3,211	-0,055	0,139	-0,414	0,679	0,995
miR-2355-3p	49,637	0,571	0,316	1,818	0,069	0,853

miR-2355-5p	2,275	0,065	0,104	0,826	NA	NA
miR-23a-3p	266375,256	0,124	0,159	0,777	0,437	0,995
miR-23a-5p	1,626	0,082	0,104	1,731	0,083	0,884
miR-23b-3p	179841,856	0,032	0,161	0,197	0,844	0,995
miR-23b-5p	15,564	0,216	0,279	0,782	0,434	0,995
miR-23c-3p	96327,534	0,253	0,143	1,766	0,077	0,853
miR-24-1-3p	14384,382	0,148	0,196	0,756	0,449	0,995
miR-24-1-5p	33,507	-0,112	0,311	-0,360	0,719	0,995
miR-24-2-3p	34,848	-0,126	0,316	-0,397	0,691	0,995
miR-24-2-5p	86,161	-0,328	0,315	-1,042	0,298	0,987
miR-24-3p	30310,039	0,286	0,173	1,659	0,097	0,892
miR-2467-3p	0,000	NA	NA	NA	NA	NA
miR-25-3p	2088,387	0,150	0,169	0,886	0,376	0,995
miR-25-5p	23,213	0,036	0,228	0,159	0,874	0,995
miR-2682-5p	2,760	0,091	0,109	2,010	0,044	0,845
miR-26a-1-5p	6,149	0,079	0,190	0,424	0,672	0,995
miR-26a-2-3p	7,396	0,016	0,161	0,097	0,923	0,995
miR-26a-2-5p	1159,400	0,096	0,175	0,549	0,583	0,995
miR-26a-5p	18396,596	0,114	0,140	0,810	0,418	0,995
miR-26b-3p	10,097	-0,008	0,260	-0,030	0,976	0,995
miR-26b-5p	4231,558	-0,051	0,178	-0,286	0,775	0,995
miR-27a-3p	17732,012	-0,262	0,166	-1,576	0,115	0,894
miR-27a-5p	35,292	0,586	0,315	1,884	0,060	0,853
miR-27b-3p	34366,159	-0,156	0,188	-0,829	0,407	0,995
miR-27b-5p	355,889	-0,074	0,213	-0,347	0,728	0,995
miR-28-3p	2481,200	0,175	0,202	0,865	0,387	0,995
miR-28-5p	3269,634	0,064	0,140	0,456	0,649	0,995
miR-2861-3p	9,529	0,000	0,193	-0,001	0,999	0,999
miR-296-3p	35,555	0,015	0,237	0,063	0,950	0,995
miR-299-5p	15,760	0,082	0,201	0,412	0,681	0,995
miR-29a-3p	337,599	-0,334	0,251	-1,330	0,184	0,894
miR-29b-1-3p	9,753	0,140	0,173	0,906	0,365	0,995
miR-29b-1-5p	124,903	-0,087	0,306	-0,286	0,775	0,995
miR-29b-2-5p	5,599	-0,033	0,142	-0,232	0,816	0,995

miR-29b-3p	3,585	0,020	0,118	0,173	0,863	0,995
miR-29c-3p	13,981	-0,201	0,219	-0,974	0,330	0,995
miR-29c-5p	14,545	-0,016	0,211	-0,074	0,941	0,995
miR-301a-3p	121,777	0,027	0,311	0,087	0,931	0,995
miR-301b-3p	7,249	0,146	0,194	0,799	0,424	0,995
miR-302a-3p	38,345	0,168	0,308	0,548	0,583	0,995
miR-302a-5p	13,168	0,043	0,212	0,204	0,839	0,995
miR-302b-3p	189,595	-0,256	0,261	-0,980	0,327	0,995
miR-302c-3p	16,792	0,167	0,221	0,784	0,433	0,995
miR-302c-5p	2,820	0,073	0,104	1,079	NA	NA
miR-302d-3p	15,149	0,013	0,217	0,061	0,951	0,995
miR-3064-5p	12,075	0,086	0,263	0,328	0,743	0,995
miR-3074-3p	1,666	0,037	0,104	0,386	0,699	0,995
miR-30a-3p	894,045	0,147	0,194	0,759	0,448	0,995
miR-30a-5p	158,218	0,725	0,305	2,483	0,013	0,470
miR-30b-3p	43,279	0,411	0,311	1,331	0,183	0,894
miR-30b-5p	309,489	0,267	0,243	1,096	0,273	0,967
miR-30c-1-3p	75,714	-0,427	0,313	-1,369	0,171	0,894
miR-30c-1-5p	1719,456	0,218	0,239	0,915	0,360	0,995
miR-30c-2-3p	70,234	-0,020	0,314	-0,065	0,948	0,995
miR-30c-5p	185,818	0,468	0,296	1,581	0,114	0,894
miR-30d-3p	16,533	-0,020	0,282	-0,071	0,944	0,995
miR-30d-5p	4409,055	0,076	0,150	0,505	0,614	0,995
miR-30e-3p	1028,608	0,139	0,183	0,760	0,447	0,995
miR-30e-5p	1207,969	-0,276	0,246	-1,121	0,262	0,954
miR-3117-3p	2,526	0,084	0,105	1,911	0,056	0,851
miR-3123-3p	31,374	-0,194	0,241	-0,830	0,407	0,995
miR-3124-5p	253,275	0,456	0,302	1,508	0,131	0,894
miR-3126-5p	8,777	0,037	0,263	0,141	0,888	0,995
miR-3129-5p	4,794	0,027	0,125	0,217	0,828	0,995
miR-3132-3p	1,304	0,082	0,104	1,649	0,099	0,892
miR-3132-5p	1,930	-0,063	0,104	-0,790	0,430	0,995
miR-3138-3p	3,341	0,060	0,118	0,575	0,565	0,995
miR-31-3p	8,937	0,131	0,194	0,706	0,480	0,995

miR-3140-3p	0,558	-0,071	0,103	-1,043	0,297	0,987
miR-3154-5p	0,000	NA	NA	NA	NA	NA
miR-3159-3p	1,951	0,083	0,104	1,799	0,072	0,853
miR-31-5p	13148,805	-0,141	0,196	-0,720	0,471	0,995
miR-3162-3p	4,423	0,054	0,145	0,386	0,699	0,995
miR-3162-5p	0,946	0,082	0,104	1,530	0,126	0,894
miR-3166-5p	3,235	0,128	0,129	2,130	0,033	0,822
miR-3175-5p	2,680	-0,010	0,110	-0,088	0,930	0,995
miR-3176-5p	4,248	-0,219	0,162	-3,133	0,002	0,181
miR-3177-3p	20,735	0,282	0,282	1,019	0,308	0,987
miR-3178-5p	155,997	-0,119	0,312	-0,383	0,702	0,995
miR-3180-3-3p	9,924	0,236	0,220	1,168	0,243	0,954
miR-3180-3-5p	18,091	-0,024	0,218	-0,110	0,912	0,995
miR-3180-3p	12,964	0,003	0,214	0,012	0,990	0,995
miR-3180-5-3p	1,946	0,004	0,104	0,043	0,966	0,995
miR-3181-3p	3,386	-0,018	0,116	-0,159	0,874	0,995
miR-3183-3p	13,598	-0,127	0,215	-0,605	0,545	0,995
miR-3187-3p	0,158	-0,075	0,099	-0,838	0,402	0,995
miR-3190-3p	0,974	-0,068	0,104	-0,902	0,367	0,995
miR-3193-5p	9,353	-0,072	0,167	-0,443	0,658	0,995
miR-3194-3p	0,683	-0,081	0,103	-1,385	0,166	0,894
miR-3195-3p	7,600	0,156	0,157	1,300	0,194	0,896
miR-3195-5p	18,301	0,099	0,287	0,346	0,729	0,995
miR-3196-3p	0,000	NA	NA	NA	NA	NA
miR-3196-5p	6,936	0,056	0,156	0,370	0,711	0,995
miR-3198-2-3p	8,491	-0,249	0,206	-1,395	0,163	0,894
miR-3199-1-5p	5,237	-0,113	0,167	-0,736	0,462	0,995
miR-3200-3p	181,807	-0,021	0,274	-0,075	0,940	0,995
miR-3200-5p	9,751	0,101	0,198	0,524	0,600	0,995
miR-3201-3p	3,613	-0,109	0,118	-2,264	0,024	0,715
miR-320a-3p	872,656	0,167	0,247	0,677	0,498	0,995
miR-320a-5p	0,598	0,081	0,103	1,360	0,174	0,894
miR-320b-1-3p	502,857	0,391	0,275	1,423	0,155	0,894
miR-320b-2-3p	15,345	0,433	0,232	2,386	NA	NA

miR-320c-1-3p	25,456	0,050	0,285	0,176	0,860	0,995
miR-320d-1-3p	13,850	-0,010	0,240	-0,043	0,966	0,995
miR-320e-3p	6,256	0,172	0,165	1,336	NA	NA
miR-323a-3p	54,598	-0,098	0,315	-0,313	0,754	0,995
miR-323a-5p	0,000	NA	NA	NA	NA	NA
miR-323b-3p	45,702	-0,045	0,301	-0,151	0,880	0,995
miR-32-3p	52,095	-0,435	0,305	-1,449	0,147	0,894
miR-324-3p	3,975	0,057	0,122	0,513	0,608	0,995
miR-324-5p	439,462	-0,101	0,243	-0,417	0,676	0,995
miR-32-5p	3,285	0,050	0,116	0,463	0,643	0,995
miR-326	4,745	-0,009	0,124	-0,070	0,945	0,995
miR-326-3p	3,069	0,037	0,104	0,376	NA	NA
miR-328-3p	32,507	0,141	0,316	0,448	0,654	0,995
miR-328-5p	0,847	-0,075	0,103	-1,205	0,228	0,948
miR-329-1-3p	4,203	0,121	0,125	2,470	0,014	0,470
miR-330-3p	113,064	-0,020	0,301	-0,067	0,947	0,995
miR-330-5p	16,361	0,047	0,209	0,228	0,820	0,995
miR-331-3p	271,082	0,104	0,268	0,388	0,698	0,995
miR-331-5p	25,412	-0,020	0,288	-0,071	0,944	0,995
miR-335-3p	296,154	-0,057	0,259	-0,219	0,827	0,995
miR-335-5p	6325,425	-0,148	0,177	-0,837	0,403	0,995
miR-337-3p	7,270	0,098	0,162	0,644	0,520	0,995
miR-337-5p	12,961	-0,218	0,220	-1,058	0,290	0,977
miR-338-3p	11,518	0,182	0,213	0,902	0,367	0,995
miR-338-5p	8,960	0,189	0,176	1,315	0,188	0,894
miR-339-3p	163,323	-0,027	0,291	-0,091	0,927	0,995
miR-339-5p	39,157	-0,108	0,309	-0,350	0,727	0,995
miR-33a-3p	5,005	0,020	0,126	0,163	0,870	0,995
miR-33a-5p	0,342	-0,079	0,102	-1,127	0,260	0,954
miR-33b-3p	53,582	0,293	0,316	0,929	0,353	0,995
miR-340-3p	83,873	-0,164	0,309	-0,530	0,596	0,995
miR-340-5p	173,821	-0,440	0,309	-1,428	0,153	0,894
miR-342-3p	2020,902	0,005	0,177	0,029	0,977	0,995
miR-342-5p	21,365	0,060	0,291	0,207	0,836	0,995

miR-345-3p	0,000	NA	NA	NA	NA	NA
miR-345-5p	108,974	-0,001	0,308	-0,004	0,997	0,998
miR-346	15,484	-0,378	0,285	-1,359	0,174	0,894
miR-346-3p	12,537	0,037	0,170	0,220	NA	NA
miR-34a-3p	12,352	0,117	0,213	0,560	0,575	0,995
miR-34a-5p	352,469	0,079	0,219	0,360	0,719	0,995
miR-34b-3p	2,802	0,068	0,104	0,914	NA	NA
miR-34c-5p	14,839	0,123	0,222	0,564	0,573	0,995
miR-3605-3p	46,762	-0,128	0,305	-0,421	0,674	0,995
miR-3605-5p	26,180	0,063	0,299	0,210	0,833	0,995
miR-3609-3p	3,191	-0,060	0,104	-0,713	NA	NA
miR-3610-3p	1,533	-0,052	0,104	-0,588	0,556	0,995
miR-3612-5p	4,302	0,102	0,126	1,115	0,265	0,954
miR-3613-3p	7,865	0,110	0,196	0,580	0,562	0,995
miR-3613-5p	4,424	-0,043	0,124	-0,364	0,716	0,995
miR-361-3p	4,163	-0,210	0,159	-3,072	0,002	0,200
miR-3614-3p	4,551	0,084	0,126	0,800	0,424	0,995
miR-3615-3p	4,823	0,082	0,151	0,582	0,560	0,995
miR-3615-5p	8,116	-0,066	0,176	-0,379	NA	NA
miR-361-5p	5900,057	0,121	0,155	0,785	0,432	0,995
miR-3616-5p	0,679	0,081	0,103	1,407	0,159	0,894
miR-3617-5p	5,080	0,120	0,133	1,313	0,189	0,894
miR-3619-3p	0,621	0,081	0,103	1,374	0,169	0,894
miR-3620-3p	3,867	0,024	0,104	0,233	NA	NA
miR-3622b-5p	9,234	0,214	0,179	1,547	0,122	0,894
miR-362-3p	6,843	-0,096	0,137	-0,820	0,412	0,995
miR-362-5p	1087,514	0,183	0,205	0,894	0,371	0,995
miR-363-3p	39,955	-0,019	0,316	-0,061	0,951	0,995
miR-3648-2-3p	21,298	0,260	0,303	0,862	0,388	0,995
miR-3648-2-5p	18,654	0,227	0,299	0,764	0,445	0,995
miR-3652-5p	0,462	0,080	0,103	1,263	0,206	0,903
miR-365a-3p	151,036	0,181	0,278	0,652	0,514	0,995
miR-365a-5p	0,000	NA	NA	NA	NA	NA
miR-365b-3p	491,712	-0,028	0,214	-0,133	0,894	0,995

miR-365b-5p	42,736	0,243	0,308	0,791	0,429	0,995
miR-3663-3p	23,346	0,346	0,285	1,242	0,214	0,930
miR-3665-5p	127,151	0,065	0,316	0,205	0,838	0,995
miR-3679-5p	4,990	-0,084	0,152	-0,589	0,556	0,995
miR-3689b-3p	0,287	-0,078	0,102	-1,063	0,288	0,974
miR-3691-5p	4,059	0,178	0,156	1,719	0,086	0,892
miR-369-3p	24,492	-0,166	0,303	-0,549	0,583	0,995
miR-369-5p	67,693	-0,006	0,314	-0,020	0,984	0,995
miR-370-3p	2,251	-0,034	0,104	-0,349	0,727	0,995
miR-374a-3p	70,640	-0,532	0,315	-1,694	0,090	0,892
miR-374a-5p	75,136	-0,296	0,311	-0,951	0,342	0,995
miR-374b-3p	16,841	-0,034	0,215	-0,159	0,874	0,995
miR-374b-5p	1703,689	0,036	0,193	0,188	0,851	0,995
miR-374c-5p	7,046	0,162	0,200	0,862	0,389	0,995
miR-375-3p	32,532	0,067	0,278	0,241	0,809	0,995
miR-376a-2-3p	7,540	-0,100	0,188	-0,548	0,584	0,995
miR-376a-3p	13,927	0,250	0,209	1,361	0,174	0,894
miR-376b-3p	6,420	-0,003	0,152	-0,018	0,985	0,995
miR-376c-3p	225,150	-0,124	0,294	-0,422	0,673	0,995
miR-378a-3p	5110,464	0,084	0,139	0,604	0,546	0,995
miR-378a-5p	30,005	0,047	0,225	0,208	0,836	0,995
miR-378c-5p	38,501	-0,178	0,303	-0,587	0,557	0,995
miR-378d-1-3p	19,521	-0,047	0,295	-0,160	0,873	0,995
miR-378e-5p	10,049	0,172	0,168	1,266	0,206	0,903
miR-378g-5p	18,347	0,199	0,162	1,961	NA	NA
miR-378i-5p	14,890	-0,159	0,245	-0,662	0,508	0,995
miR-379-5p	3,506	0,066	0,164	0,410	0,682	0,995
miR-380-3p	3,085	0,041	0,114	0,383	0,702	0,995
miR-381-3p	0,909	0,001	0,104	0,013	0,990	0,995
miR-382-5p	65,375	-0,084	0,316	-0,266	0,790	0,995
miR-3907-3p	2,816	-0,048	0,107	-0,495	NA	NA
miR-3909	10,796	-0,153	0,265	-0,582	0,561	0,995
miR-3909-3p	3,064	-0,065	0,137	-0,508	0,611	0,995
miR-3909-5p	4,892	-0,051	0,108	-0,533	NA	NA

miR-3916-5p	5,699	-0,150	0,137	-2,824	0,005	0,372
miR-3919-3p	76,835	-0,045	0,316	-0,143	0,886	0,995
miR-3928-3p	7,278	-0,193	0,164	-1,729	0,084	0,884
miR-3929-3p	13,644	0,083	0,214	0,390	0,696	0,995
miR-3934-3p	1,244	0,076	0,104	1,188	0,235	0,948
miR-3934-5p	0,712	0,081	0,103	1,424	0,154	0,894
miR-3940-3p	0,761	0,081	0,103	1,449	0,147	0,894
miR-3940-5p	0,971	-0,051	0,104	-0,569	0,569	0,995
miR-3944-5p	45,745	0,096	0,266	0,360	0,719	0,995
miR-3960-3p	33,250	-0,504	0,309	-1,652	0,099	0,892
miR-3960-5p	2,247	-0,011	0,104	-0,104	0,917	0,995
miR-3976-5p	0,000	NA	NA	NA	NA	NA
miR-409-3p	38,190	-0,127	0,315	-0,404	0,686	0,995
miR-409-5p	33,242	-0,199	0,311	-0,641	0,521	0,995
miR-411-3p	9,023	-0,103	0,260	-0,397	0,691	0,995
miR-411-5p	18,112	-0,019	0,278	-0,068	0,945	0,995
miR-412-5p	0,995	-0,032	0,104	-0,326	0,744	0,995
miR-421	354,859	-0,065	0,239	-0,271	0,786	0,995
miR-421-3p	113,587	0,213	0,312	0,684	0,494	0,995
miR-423-3p	65,819	0,142	0,312	0,455	0,649	0,995
miR-423-5p	2259,241	-0,215	0,206	-1,040	0,298	0,987
miR-424-3p	112,164	0,179	0,301	0,596	0,551	0,995
miR-424-5p	1407,878	-0,312	0,206	-1,515	0,130	0,894
miR-425-3p	579,484	0,295	0,192	1,533	0,125	0,894
miR-425-5p	406,796	0,151	0,231	0,651	0,515	0,995
miR-4259-5p	74,560	-0,207	0,258	-0,818	0,413	0,995
miR-4267-5p	2,610	-0,061	0,110	-0,656	0,512	0,995
miR-4270-5p	13,156	0,008	0,202	0,038	0,969	0,995
miR-4271-3p	1,700	0,109	0,126	1,280	0,201	0,903
miR-4284-3p	4,593	-0,216	0,161	-3,163	0,002	0,181
miR-4284-5p	10,929	0,123	0,223	0,562	0,574	0,995
miR-4285-5p	6,434	0,079	0,150	0,560	0,575	0,995
miR-4286	4,600	-0,053	0,124	-0,459	0,646	0,995
miR-4286-5p	148,257	0,175	0,281	0,623	0,533	0,995

miR-4291-5p	5,518	0,143	0,134	2,776	0,006	0,398
miR-4293-5p	3,071	0,065	0,104	0,833	NA	NA
miR-4301-5p	22,465	-0,024	0,302	-0,080	0,937	0,995
miR-4306-3p	0,499	-0,080	0,103	-1,269	0,205	0,903
miR-4309-3p	3,207	0,079	0,117	0,859	0,390	0,995
miR-4311-3p	2,862	-0,083	0,104	-1,917	NA	NA
miR-4313-3p	19,287	0,128	0,265	0,485	0,628	0,995
miR-4313-5p	3,351	0,086	0,120	0,936	0,349	0,995
miR-431-5p	9,119	-0,010	0,197	-0,052	0,958	0,995
miR-4316-5p	0,000	NA	NA	NA	NA	NA
miR-4322-3p	0,709	0,075	0,103	1,164	0,244	0,954
miR-4322-5p	8,417	-0,081	0,219	-0,370	0,711	0,995
miR-432-5p	24,099	0,096	0,230	0,420	0,674	0,995
miR-4422-5p	14,703	-0,285	0,271	-1,075	0,282	0,970
miR-4423-3p	11,547	-0,112	0,202	-0,568	0,570	0,995
miR-4423-5p	1,025	-0,082	0,104	-1,536	0,125	0,894
miR-4425-3p	20,289	0,128	0,295	0,434	0,665	0,995
miR-4429-5p	0,815	0,082	0,103	1,475	0,140	0,894
miR-4437-5p	0,489	0,080	0,103	1,285	0,199	0,903
miR-4442-5p	2,386	0,104	0,117	1,823	0,068	0,853
miR-4443-5p	9,846	-0,181	0,198	-0,991	0,322	0,995
miR-4445-3p	2,683	-0,012	0,104	-0,119	0,905	0,995
miR-4449-3p	16,897	-0,047	0,215	-0,220	0,826	0,995
miR-4449-5p	7,976	-0,079	0,202	-0,398	0,691	0,995
miR-4453-3p	7,706	0,053	0,174	0,309	0,757	0,995
miR-4453-5p	0,158	-0,075	0,099	-0,838	0,402	0,995
miR-4454-3p	4,932	-0,010	0,123	-0,085	0,932	0,995
miR-4454-5p	7,844	0,209	0,246	0,872	0,383	0,995
miR-4455-5p	271,582	-0,034	0,249	-0,137	0,891	0,995
miR-4462-3p	0,000	NA	NA	NA	NA	NA
miR-4463-3p	1,189	0,082	0,104	1,615	0,106	0,892
miR-4466-5p	0,000	NA	NA	NA	NA	NA
miR-4484-3p	63,709	-0,014	0,314	-0,044	0,965	0,995
miR-4485-3p	249,195	-0,036	0,293	-0,124	0,901	0,995

miR-4485-5p	2,856	0,041	0,104	0,427	NA	NA
miR-4488	3,061	-0,038	0,104	-0,391	NA	NA
miR-4488-5p	275,972	0,267	0,294	0,911	0,363	0,995
miR-4492-3p	45,509	-0,151	0,294	-0,516	0,606	0,995
miR-4496-3p	0,526	-0,080	0,103	-1,288	0,198	0,903
miR-4497-3p	7,055	0,069	0,148	0,490	0,624	0,995
miR-4497-5p	22,882	-0,071	0,262	-0,270	0,787	0,995
miR-4498-3p	1,015	-0,003	0,104	-0,027	0,978	0,995
miR-4502-3p	1,235	0,008	0,104	0,077	0,938	0,995
miR-4506-3p	6,060	0,149	0,161	1,113	0,266	0,954
miR-4508-5p	5,703	0,031	0,171	0,181	0,856	0,995
miR-450a-1-5p	213,293	-0,206	0,277	-0,743	0,458	0,995
miR-450a-5p	243,315	0,152	0,255	0,597	0,550	0,995
miR-450b-5p	42,526	-0,587	0,316	-1,869	0,062	0,853
miR-4512-5p	1,332	0,082	0,104	1,657	0,098	0,892
miR-4516-5p	34,964	-0,360	0,313	-1,157	0,247	0,954
miR-451a-5p	0,032	0,025	0,096	0,259	0,796	0,995
miR-4521-5p	166,350	-0,067	0,279	-0,239	0,811	0,995
miR-452-3p	1,942	0,083	0,104	1,797	0,072	0,853
miR-4524b-3p	11,786	0,090	0,276	0,328	0,743	0,995
miR-452-5p	113,392	0,050	0,315	0,160	0,873	0,995
miR-454-3p	91,288	-0,324	0,301	-1,074	0,283	0,970
miR-455-3p	11531,226	0,023	0,160	0,147	0,883	0,995
miR-455-5p	2,747	0,059	0,113	0,604	0,546	0,995
miR-4633-5p	13,270	-0,079	0,200	-0,403	0,687	0,995
miR-4636	20,779	-0,137	0,293	-0,469	0,639	0,995
miR-4636-5p	17,546	0,070	0,219	0,320	0,749	0,995
miR-4642-3p	0,789	0,081	0,103	1,463	0,144	0,894
miR-4647-3p	0,900	0,072	0,104	1,034	0,301	0,987
miR-4651-5p	0,932	0,001	0,133	0,011	0,992	0,995
miR-4654-5p	2,120	0,083	0,104	1,829	NA	NA
miR-4655-3p	15,076	0,288	0,227	1,410	0,158	0,894
miR-4661-5p	3,936	-0,052	0,121	-0,457	0,648	0,995
miR-4662a-5p	10,553	-0,020	0,261	-0,077	0,939	0,995

miR-4663-3p	3,572	0,083	0,104	2,023	NA	NA
miR-4664-3p	4,678	-0,123	0,132	-1,430	0,153	0,894
miR-4665-5p	0,924	0,082	0,104	1,521	0,128	0,894
miR-4667-5p	3,565	-0,007	0,117	-0,061	0,952	0,995
miR-4669-3p	0,000	NA	NA	NA	NA	NA
miR-4671-5p	3,092	-0,061	0,114	-0,627	0,531	0,995
miR-4674-3p	0,000	NA	NA	NA	NA	NA
miR-4677-3p	6,645	-0,014	0,156	-0,092	0,927	0,995
miR-4683-3p	5,749	-0,267	0,187	-2,045	0,041	0,845
miR-4685-5p	0,000	NA	NA	NA	NA	NA
miR-4687-3p	5,777	-0,144	0,163	-1,030	0,303	0,987
miR-4687-5p	0,000	NA	NA	NA	NA	NA
miR-4701-3p	0,702	0,081	0,103	1,420	0,156	0,894
miR-4706-5p	0,000	NA	NA	NA	NA	NA
miR-4713-3p	1,075	0,029	0,104	0,293	0,769	0,995
miR-4721-3p	1,912	-0,082	0,104	-1,767	0,077	0,853
miR-4723-3p	4,302	0,082	0,125	0,782	0,434	0,995
miR-4723-5p	1,848	0,030	0,104	0,303	0,762	0,995
miR-4728-3p	0,052	0,002	0,096	0,023	0,982	0,995
miR-4728-5p	1,090	-0,078	0,104	-1,303	0,192	0,896
miR-4738-3p	6,232	0,034	0,154	0,221	0,825	0,995
miR-4739-5p	4,117	-0,121	0,124	-2,430	0,015	0,507
miR-4741-5p	4,953	-0,011	0,108	-0,107	NA	NA
miR-4742-5p	18,661	-0,502	0,295	-1,757	0,079	0,853
miR-4745-5p	1,235	-0,082	0,104	-1,605	0,108	0,892
miR-4751-5p	0,457	-0,068	0,103	-0,963	0,335	0,995
miR-4755-3p	2,560	-0,060	0,126	-0,511	0,609	0,995
miR-4757-5p	28,940	0,257	0,284	0,916	0,360	0,995
miR-4758-3p	4,897	0,023	0,147	0,158	0,874	0,995
miR-4767-3p	0,082	-0,020	0,096	-0,204	0,838	0,995
miR-4767-5p	14,593	-0,145	0,211	-0,712	0,477	0,995
miR-4769-5p	1,353	-0,048	0,104	-0,529	0,597	0,995
miR-4781-5p	2,115	0,083	0,104	1,829	0,067	0,853
miR-4784-3p	1,331	-0,082	0,104	-1,633	0,102	0,892

miR-4784-5p	0,000	NA	NA	NA	NA	NA
miR-4785-5p	1,167	0,082	0,104	1,608	0,108	0,892
miR-4786-3p	0,815	0,082	0,103	1,475	0,140	0,894
miR-4786-5p	3,214	-0,127	0,128	-2,090	0,037	0,829
miR-4787-3p	16,236	-0,049	0,219	-0,225	0,822	0,995
miR-4787-5p	14,401	-0,093	0,220	-0,427	0,670	0,995
miR-4793-3p	2,821	-0,037	0,104	-0,387	0,699	0,995
miR-4796-3p	1,630	-0,079	0,104	-1,456	0,145	0,894
miR-4800-3p	4,059	-0,004	0,167	-0,025	0,980	0,995
miR-4803-5p	7,361	0,039	0,188	0,207	0,836	0,995
miR-4804-5p	9,015	-0,068	0,166	-0,421	0,674	0,995
miR-483-3p	1178,424	-0,351	0,260	-1,351	0,177	0,894
miR-483-5p	77,752	-0,122	0,316	-0,387	0,699	0,995
miR-484	351,332	0,302	0,238	1,269	0,205	0,903
miR-484-5p	264,656	0,342	0,256	1,335	0,182	0,894
miR-485-5p	17,615	0,172	0,271	0,643	0,520	0,995
miR-486-2-5p	8,840	-0,273	0,243	-1,191	0,234	0,948
miR-486-3p	0,652	0,081	0,103	1,392	0,164	0,894
miR-486-5p	868,371	-0,052	0,206	-0,250	0,802	0,995
miR-487a-3p	10,706	-0,059	0,198	-0,300	0,764	0,995
miR-487b-3p	49,902	0,033	0,316	0,106	0,916	0,995
miR-489-3p	15,904	0,238	0,210	1,275	NA	NA
miR-490-3p	57,953	0,168	0,309	0,546	0,585	0,995
miR-490-5p	231,256	0,335	0,274	1,222	0,222	0,947
miR-491-3p	1,925	0,083	0,104	1,794	0,073	0,853
miR-491-5p	42,245	0,523	0,312	1,700	0,089	0,892
miR-493-3p	5,717	-0,055	0,151	-0,371	0,710	0,995
miR-493-5p	23,752	-0,144	0,300	-0,481	0,631	0,995
miR-494-3p	84,357	-0,107	0,316	-0,339	0,735	0,995
miR-495-3p	14,925	-0,051	0,215	-0,238	0,812	0,995
miR-497-5p	16,627	-0,190	0,291	-0,657	0,511	0,995
miR-498-3p	1,607	0,012	0,104	0,116	0,907	0,995
miR-4999-5p	2,716	0,089	0,108	1,985	0,047	0,845
miR-499a-3p	0,815	-0,081	0,103	-1,451	0,147	0,894

miR-499a-5p	2909,180	-0,120	0,254	-0,473	0,636	0,995
miR-5001-3p	0,027	0,025	0,096	0,259	0,796	0,995
miR-5001-5p	13,461	0,146	0,210	0,725	0,468	0,995
miR-5004-3p	2,288	-0,083	0,104	-1,834	0,067	0,853
miR-5006-3p	2,627	0,088	0,107	1,965	0,049	0,845
miR-500a-3p	1668,520	0,051	0,168	0,304	0,761	0,995
miR-500a-5p	15,605	0,038	0,275	0,137	0,891	0,995
miR-500b-3p	4,764	-0,064	0,158	-0,420	0,675	0,995
miR-500b-5p	19,493	0,161	0,243	0,672	0,501	0,995
miR-5010-3p	21,958	0,112	0,295	0,380	0,704	0,995
miR-5010-5p	9,388	-0,031	0,206	-0,149	0,882	0,995
miR-501-3p	825,139	0,055	0,220	0,250	0,803	0,995
miR-501-5p	38,697	0,225	0,311	0,726	0,468	0,995
miR-502-3p	599,502	0,107	0,225	0,474	0,635	0,995
miR-502-5p	6,176	0,012	0,154	0,075	0,940	0,995
miR-503-5p	274,384	0,141	0,234	0,605	0,545	0,995
miR-504-3p	4,464	0,047	0,173	0,273	0,785	0,995
miR-504-5p	21,118	-0,028	0,220	-0,126	0,899	0,995
miR-505-3p	103,362	-0,183	0,309	-0,593	0,553	0,995
miR-505-5p	320,805	-0,220	0,294	-0,749	0,454	0,995
miR-506-3p	7,859	-0,200	0,192	-1,197	0,231	0,948
miR-508-3p	0,000	NA	NA	NA	NA	NA
miR-5091-5p	1,563	0,040	0,104	0,418	0,676	0,995
miR-5092-3p	6,766	-0,101	0,133	-0,942	NA	NA
miR-5093-3p	3,051	0,081	0,104	1,715	NA	NA
miR-5100-3p	60,831	0,234	0,315	0,746	0,455	0,995
miR-513b-5p	2,944	-0,038	0,113	-0,352	0,725	0,995
miR-514a-1-5p	3,752	0,056	0,141	0,415	0,678	0,995
miR-514b-5p	0,000	NA	NA	NA	NA	NA
miR-5187-5p	3,330	0,068	0,166	0,420	0,674	0,995
miR-5191-3p	5,864	-0,060	0,152	-0,405	0,685	0,995
miR-5195-3p	9,057	0,033	0,196	0,167	0,868	0,995
miR-5197-3p	2,962	0,076	0,111	0,946	NA	NA
miR-520g-5p	0,027	0,025	0,096	0,259	0,796	0,995

miR-524-3p	4,033	0,029	0,104	0,285	NA	NA
miR-532-3p	153,005	0,043	0,300	0,143	0,886	0,995
miR-532-5p	370,140	0,390	0,222	1,760	0,078	0,853
miR-542-3p	10,977	-0,046	0,204	-0,227	0,820	0,995
miR-542-5p	33,098	0,251	0,306	0,822	0,411	0,995
miR-543-5p	11,310	-0,071	0,146	-0,511	0,609	0,995
miR-548ae-2-5p	2,307	-0,047	0,107	-0,490	0,624	0,995
miR-548am-5p	3,408	0,105	0,117	2,233	0,026	0,747
miR-548as-5p	1,897	0,083	0,104	1,788	0,074	0,853
miR-548ay-5p	3,435	-0,037	0,136	-0,274	0,784	0,995
miR-548bc-3p	2,604	-0,087	0,107	-1,929	0,054	0,845
miR-548d-1-5p	36,476	0,168	0,316	0,532	0,595	0,995
miR-548h-5-5p	9,973	0,303	0,192	2,526	0,012	0,470
miR-548k	25,357	0,283	0,292	0,980	0,327	0,995
miR-548t-3p	3,042	0,036	0,104	0,371	NA	NA
miR-548x-2-5p	2,398	-0,083	0,104	-1,851	NA	NA
miR-549-5p	0,679	0,081	0,103	1,407	0,159	0,894
miR-550a-1-5p	17,441	0,074	0,293	0,254	0,799	0,995
miR-550a-3-3p	2,539	-0,015	0,109	-0,139	0,890	0,995
miR-550a-3-5p	4,749	-0,145	0,166	-1,016	0,310	0,987
miR-551b-3p	6,822	0,078	0,190	0,419	0,675	0,995
miR-551b-5p	12,511	-0,012	0,207	-0,056	0,955	0,995
miR-556-3p	6,249	-0,073	0,189	-0,392	0,695	0,995
miR-556-5p	61,308	-0,211	0,255	-0,847	0,397	0,995
miR-5572-5p	1,509	-0,017	0,104	-0,162	0,871	0,995
miR-5584-5p	1,983	0,100	0,114	1,970	0,049	0,845
miR-5585-3p	3,770	-0,015	0,119	-0,125	0,901	0,995
miR-5585-5p	0,289	-0,078	0,102	-1,065	0,287	0,974
miR-5587-3p	3,738	0,111	0,120	2,329	0,020	0,622
miR-564-3p	3,177	-0,031	0,114	-0,279	0,780	0,995
miR-5684-3p	0,697	0,001	0,103	0,011	0,992	0,995
miR-5684-5p	8,904	0,051	0,165	0,317	0,752	0,995
miR-5689-5p	2,111	0,083	0,104	1,828	0,068	0,853
miR-5694-3p	0,425	-0,080	0,103	-1,208	0,227	0,948

miR-5699-3p	69,698	0,375	0,313	1,208	0,227	0,948
miR-5699-5p	5,675	-0,069	0,112	-0,762	NA	NA
miR-570-3p	4,122	-0,044	0,104	-0,468	NA	NA
miR-5708-3p	6,004	0,033	0,112	0,306	NA	NA
miR-572-3p	1,940	0,044	0,104	0,464	0,642	0,995
miR-572-5p	1,482	0,023	0,104	0,231	0,818	0,995
miR-574-3p	1466,474	0,125	0,203	0,615	0,538	0,995
miR-574-5p	138,602	0,218	0,282	0,773	0,439	0,995
miR-576-3p	25,378	0,158	0,229	0,709	0,478	0,995
miR-576-5p	23,285	-0,208	0,234	-0,924	0,356	0,995
miR-577-5p	0,000	NA	NA	NA	NA	NA
miR-5787-5p	9,214	-0,122	0,167	-0,799	0,424	0,995
miR-582-5p	20,084	-0,207	0,229	-0,948	0,343	0,995
miR-584-5p	58,908	0,301	0,314	0,959	0,338	0,995
miR-585-3p	0,000	NA	NA	NA	NA	NA
miR-585-5p	26,306	0,185	0,297	0,627	0,530	0,995
miR-589-3p	22,818	-0,148	0,218	-0,698	0,485	0,995
miR-589-5p	152,625	0,075	0,266	0,281	0,779	0,995
miR-590-3p	53,057	-0,161	0,314	-0,514	0,607	0,995
miR-591-3p	4,037	-0,016	0,120	-0,137	0,891	0,995
miR-591-5p	14,346	-0,034	0,177	-0,191	0,849	0,995
miR-592-5p	11,290	-0,055	0,137	-0,419	NA	NA
miR-598-3p	234,258	0,078	0,274	0,286	0,775	0,995
miR-598-5p	2,470	0,024	0,104	0,237	0,813	0,995
miR-601-5p	9,373	0,033	0,166	0,201	0,841	0,995
miR-602-3p	0,380	0,080	0,102	1,191	0,234	0,948
miR-605-3p	14,359	-0,028	0,177	-0,159	0,874	0,995
miR-6068-3p	4,813	0,087	0,128	0,806	0,420	0,995
miR-6073-3p	85,837	0,041	0,316	0,128	0,898	0,995
miR-6075-3p	14,786	0,197	0,260	0,771	0,441	0,995
miR-6076-3p	11,908	-0,130	0,170	-0,846	0,397	0,995
miR-6082-5p	5,953	0,011	0,129	0,088	0,930	0,995
miR-6083-5p	71,312	0,063	0,313	0,201	0,841	0,995
miR-6084-5p	9,141	0,060	0,140	0,446	0,655	0,995

miR-6088-5p	8,115	0,377	0,202	4,324	0,000	0,007
miR-6089-2-3p	4,912	0,135	0,131	2,657	0,008	0,470
miR-6090-5p	7,608	-0,029	0,150	-0,195	0,845	0,995
miR-6124-5p	1,853	0,074	0,104	1,141	0,254	0,954
miR-6125-3p	1,000	0,068	0,104	0,915	0,360	0,995
miR-6126-5p	53,003	-0,056	0,316	-0,176	0,860	0,995
miR-6127-3p	4,050	0,056	0,119	0,519	NA	NA
miR-6130-3p	0,788	-0,081	0,103	-1,439	0,150	0,894
miR-6131-3p	6,888	0,176	0,157	1,638	0,101	0,892
miR-614-3p	0,000	NA	NA	NA	NA	NA
miR-615-3p	5,860	0,038	0,165	0,231	0,817	0,995
miR-616-3p	2,451	-0,073	0,110	-0,871	0,384	0,995
miR-618-3p	2,168	-0,037	0,119	-0,320	0,749	0,995
miR-619-5p	24,853	0,330	0,300	1,115	0,265	0,954
miR-622-3p	6,363	-0,081	0,131	-0,698	NA	NA
miR-622-5p	7,636	0,041	0,155	0,269	0,788	0,995
miR-624-5p	14,710	0,058	0,214	0,272	0,786	0,995
miR-625-3p	6,231	-0,027	0,153	-0,180	0,857	0,995
miR-625-5p	67,117	0,170	0,310	0,549	0,583	0,995
miR-627-5p	3,180	0,211	0,169	1,823	0,068	0,853
miR-628-3p	583,943	0,189	0,259	0,730	0,465	0,995
miR-628-5p	20,360	0,262	0,293	0,901	0,367	0,995
miR-629-3p	5,572	-0,110	0,133	-1,088	0,277	0,970
miR-629-5p	28,823	-0,083	0,311	-0,266	0,790	0,995
miR-631-5p	9,785	0,071	0,199	0,361	0,718	0,995
miR-638-5p	17,705	-0,037	0,231	-0,161	0,872	0,995
miR-639-5p	57,339	0,773	0,316	2,480	0,013	0,470
miR-641	4,425	0,013	0,122	0,109	0,914	0,995
miR-641-5p	47,151	0,025	0,315	0,079	0,937	0,995
miR-642a-3p	22,291	-0,094	0,284	-0,330	0,741	0,995
miR-642a-5p	9,796	-0,106	0,167	-0,682	0,495	0,995
miR-642b-3p	15,318	-0,169	0,271	-0,627	0,531	0,995
miR-646-3p	14,434	0,147	0,176	0,933	0,351	0,995
miR-6501-3p	7,393	-0,040	0,172	-0,235	0,814	0,995

miR-6505-5p	15,814	-0,089	0,213	-0,426	NA	NA
miR-6507-5p	0,552	-0,081	0,103	-1,306	0,192	0,896
miR-6508-3p	0,000	NA	NA	NA	NA	NA
miR-6511a-4-5p	1,692	-0,045	0,104	-0,478	0,632	0,995
miR-6511b-1-3p	7,754	-0,016	0,135	-0,123	0,902	0,995
miR-6514-5p	6,270	0,050	0,131	0,398	0,691	0,995
miR-6515-5p	2,575	0,025	0,109	0,238	0,812	0,995
miR-651-5p	4,093	0,050	0,121	0,438	0,662	0,995
miR-6516-3p	0,972	-0,082	0,104	-1,517	0,129	0,894
miR-6516-5p	8,388	0,043	0,164	0,265	0,791	0,995
miR-652-3p	4363,448	0,107	0,172	0,626	0,531	0,995
miR-652-5p	26,668	-0,175	0,291	-0,602	0,547	0,995
miR-6529-5p	4,291	0,094	0,126	0,958	0,338	0,995
miR-653-3p	9,560	0,202	0,200	1,120	0,263	0,954
miR-653-5p	6,531	-0,131	0,140	-1,328	0,184	0,894
miR-654-3p	35,255	0,179	0,315	0,569	0,569	0,995
miR-654-5p	0,263	-0,078	0,101	-1,029	0,303	0,987
miR-657-3p	9,046	0,005	0,139	0,035	0,972	0,995
miR-658-3p	3,892	0,051	0,121	0,449	0,653	0,995
miR-660-3p	25,361	-0,103	0,287	-0,358	0,720	0,995
miR-660-5p	44,220	-0,062	0,316	-0,197	0,844	0,995
miR-663a-3p	234,065	0,163	0,273	0,597	0,551	0,995
miR-663a-5p	13,254	-0,197	0,166	-1,691	0,091	0,892
miR-663b-3p	20,965	-0,162	0,228	-0,732	0,464	0,995
miR-664-3p	70,536	0,150	0,315	0,477	0,634	0,995
miR-664-5p	50,527	-0,318	0,316	-1,009	0,313	0,994
miR-664a-3p	45,626	0,296	0,316	0,937	0,349	0,995
miR-664b-3p	1,885	0,038	0,104	0,393	0,694	0,995
miR-664b-5p	1,485	0,019	0,104	0,186	0,852	0,995
miR-665-3p	0,598	0,081	0,103	1,360	0,174	0,894
miR-668-3p	1,846	0,078	0,104	1,315	0,189	0,894
miR-668-5p	4,661	-0,128	0,128	-2,547	0,011	0,470
miR-671-3p	48,942	-0,254	0,315	-0,806	0,420	0,995
miR-671-5p	99,104	0,101	0,314	0,322	0,748	0,995

miR-6716-5p	1,202	-0,001	0,115	-0,009	0,993	0,995
miR-6722-5p	20,015	0,213	0,288	0,745	0,456	0,995
miR-6724-4-5p	1,131	0,039	0,104	0,410	0,682	0,995
miR-6729-3p	12,532	-0,091	0,202	-0,461	0,645	0,995
miR-6731-5p	0,519	0,081	0,103	1,307	0,191	0,896
miR-6734-5p	5,096	0,019	0,148	0,127	0,899	0,995
miR-6735-5p	3,828	0,069	0,122	0,653	0,513	0,995
miR-6739-5p	4,189	-0,083	0,104	-2,058	NA	NA
miR-6740-5p	0,000	NA	NA	NA	NA	NA
miR-6741-3p	0,000	NA	NA	NA	NA	NA
miR-6743-3p	7,526	0,076	0,192	0,405	0,686	0,995
miR-6746-5p	0,864	0,051	0,104	0,572	0,568	0,995
miR-6748-3p	3,479	0,069	0,132	0,566	0,571	0,995
miR-6750-3p	2,673	-0,007	0,104	-0,069	0,945	0,995
miR-6752-5p	0,630	0,081	0,103	1,379	0,168	0,894
miR-6753-3p	1,542	0,082	0,104	1,712	0,087	0,892
miR-6753-5p	0,000	NA	NA	NA	NA	NA
miR-675-3p	28,278	-0,257	0,309	-0,835	0,404	0,995
miR-675-5p	315,021	0,487	0,265	1,839	0,066	0,853
miR-6756-3p	0,604	-0,081	0,103	-1,340	0,180	0,894
miR-6757-5p	3,006	-0,097	0,112	-2,080	0,038	0,829
miR-6758-5p	0,000	NA	NA	NA	NA	NA
miR-6760-5p	0,025	-0,020	0,096	-0,213	0,831	0,995
miR-6763-3p	9,883	0,045	0,198	0,228	0,819	0,995
miR-676-3p	2,849	-0,090	0,108	-1,989	0,047	0,845
miR-6765-3p	0,030	-0,020	0,096	-0,213	0,831	0,995
miR-6766-5p	5,771	0,083	0,155	0,570	0,569	0,995
miR-6768-5p	0,057	0,002	0,096	0,023	0,982	0,995
miR-6769a-3p	1,062	-0,019	0,104	-0,185	0,853	0,995
miR-6770-3-3p	3,134	-0,098	0,113	-2,106	0,035	0,829
miR-6770-3-5p	6,823	-0,124	0,194	-0,668	0,504	0,995
miR-6773-5p	7,434	-0,030	0,160	-0,191	0,849	0,995
miR-6774-5p	1,245	0,082	0,104	1,632	0,103	0,892
miR-6777-5p	2,645	0,067	0,125	0,599	0,549	0,995

miR-6778-5p	1,005	0,082	0,104	1,553	0,121	0,894
miR-6780a-5p	0,079	-0,064	0,096	-0,579	0,562	0,995
miR-6784-3p	4,979	-0,082	0,127	-0,760	0,447	0,995
miR-6785-5p	2,758	0,089	0,108	1,997	0,046	0,845
miR-6786-5p	60,102	0,838	0,316	2,688	0,007	0,470
miR-6789-3p	32,999	-0,294	0,292	-1,020	0,308	0,987
miR-6789-5p	0,665	-0,081	0,103	-1,376	0,169	0,894
miR-6790-3p	2,041	-0,049	0,104	-0,534	0,594	0,995
miR-6791-3p	1,266	0,082	0,104	1,638	0,101	0,892
miR-6793-5p	1,794	0,082	0,104	1,768	0,077	0,853
miR-6796-3p	4,611	0,083	0,104	2,117	NA	NA
miR-6797-3p	2,219	0,002	0,104	0,022	0,982	0,995
miR-6801-3p	3,287	0,047	0,111	0,459	NA	NA
miR-6805-3p	1,989	0,051	0,104	0,570	0,568	0,995
miR-6809-5p	3,016	0,050	0,133	0,387	0,699	0,995
miR-6815-5p	16,518	-0,266	0,271	-1,002	0,316	0,995
miR-6818-5p	2,314	0,047	0,107	0,487	0,626	0,995
miR-6819-3p	0,000	NA	NA	NA	NA	NA
miR-6820-3p	5,366	-0,004	0,166	-0,024	0,981	0,995
miR-6832-5p	5,250	0,125	0,145	1,095	0,274	0,967
miR-6833-5p	0,000	NA	NA	NA	NA	NA
miR-6835-5p	10,948	0,119	0,203	0,605	0,545	0,995
miR-6836-3p	0,000	NA	NA	NA	NA	NA
miR-6838-5p	1,327	0,082	0,104	1,656	0,098	0,892
miR-6843-5p	21,505	0,259	0,221	1,295	0,195	0,896
miR-6845-3p	10,824	0,277	0,199	1,766	0,077	0,853
miR-6847-5p	0,184	-0,076	0,100	-0,896	0,370	0,995
miR-6848-5p	2,856	0,002	0,111	0,020	0,984	0,995
miR-6858-3p	0,000	NA	NA	NA	NA	NA
miR-6860-5p	1,797	-0,082	0,104	-1,745	NA	NA
miR-6861-3p	0,342	-0,079	0,102	-1,127	0,260	0,954
miR-6865-5p	1,135	0,082	0,104	1,598	0,110	0,892
miR-6869-5p	4,575	-0,115	0,142	-0,988	0,323	0,995
miR-6870-5p	1,031	0,082	0,104	1,562	0,118	0,894

miR-6872-3p	6,242	0,080	0,134	0,670	0,503	0,995
miR-6875-5p	3,288	-0,103	0,115	-2,173	0,030	0,778
miR-6876-5p	2,031	0,001	0,104	0,013	0,990	0,995
miR-6877-5p	5,889	0,287	0,182	3,721	0,000	0,037
miR-6879-5p	29,524	0,267	0,298	0,902	0,367	0,995
miR-6880-5p	0,245	0,078	0,101	1,026	0,305	0,987
miR-6885-3p	25,309	0,283	0,239	1,267	0,205	0,903
miR-6885-5p	9,049	-0,259	0,182	-2,211	0,027	0,747
miR-6891-5p	6,131	0,071	0,132	0,587	0,557	0,995
miR-6894-5p	0,000	NA	NA	NA	NA	NA
miR-6895-3p	19,780	0,128	0,180	0,759	0,448	0,995
miR-6895-5p	12,247	0,042	0,204	0,204	0,838	0,995
miR-708-5p	100,403	0,076	0,315	0,242	0,809	0,995
miR-7108-3p	18,038	0,077	0,192	0,408	0,683	0,995
miR-7108-5p	1,417	0,009	0,104	0,087	0,931	0,995
miR-7109-3p	13,047	0,195	0,158	2,179	0,029	0,778
miR-7109-5p	3,541	0,077	0,104	1,242	NA	NA
miR-7113-5p	5,184	0,253	0,176	2,538	0,011	0,470
miR-711-5p	1,889	0,044	0,113	0,424	0,671	0,995
miR-7-1-3p	6,249	0,089	0,147	0,661	0,509	0,995
miR-7150-3p	3,937	0,148	0,149	1,372	0,170	0,894
miR-7151-3p	3,747	0,084	0,122	0,857	0,392	0,995
miR-7157-5p	5,264	-0,065	0,150	-0,449	0,654	0,995
miR-7-1-5p	21,201	-0,058	0,227	-0,257	0,797	0,995
miR-7160-3p	38,304	-0,038	0,303	-0,125	0,901	0,995
miR-7161-3p	0,408	0,080	0,102	1,217	0,224	0,947
miR-7162-5p	4,388	0,128	0,128	2,546	0,011	0,470
miR-718-3p	24,432	0,114	0,301	0,380	0,704	0,995
miR-718-5p	14,721	0,180	0,208	0,920	0,358	0,995
miR-7-3-5p	3,898	-0,080	0,196	-0,414	0,679	0,995
miR-744-3p	1,196	0,082	0,104	1,617	0,106	0,892
miR-744-5p	867,019	0,208	0,209	0,994	0,320	0,995
miR-758-3p	3,342	0,114	0,141	0,999	0,318	0,995
miR-7-5p	33,236	0,010	0,313	0,031	0,975	0,995

miR-760-3p	0,756	0,081	0,103	1,447	0,148	0,894
miR-760-5p	14,605	0,265	0,193	1,820	0,069	0,853
miR-762-3p	7,220	0,341	0,195	4,095	0,000	0,011
miR-762-5p	3,249	0,054	0,116	0,511	0,610	0,995
miR-765-3p	80,960	0,186	0,315	0,590	0,555	0,995
miR-766-3p	31,567	0,325	0,244	1,447	0,148	0,894
miR-766-5p	12,136	0,052	0,172	0,306	0,760	0,995
miR-769-3p	9,715	-0,182	0,149	-3,332	NA	NA
miR-769-5p	40,772	0,055	0,316	0,175	0,861	0,995
miR-7704-5p	10,514	-0,118	0,169	-0,752	0,452	0,995
miR-7706	14,935	-0,025	0,207	-0,123	0,902	0,995
miR-7706-3p	19,727	0,398	0,287	1,424	0,154	0,894
miR-7706-5p	5,125	0,010	0,126	0,083	0,934	0,995
miR-7846-3p	40,666	-0,415	0,311	-1,345	0,179	0,894
miR-7847-3p	2,111	0,029	0,161	0,179	0,858	0,995
miR-7851-3p	5,263	-0,002	0,149	-0,014	0,988	0,995
miR-7851-5p	3,602	-0,050	0,119	-0,455	0,649	0,995
miR-7975-3p	47,865	-0,379	0,304	-1,263	0,207	0,903
miR-7977-5p	263,239	0,282	0,273	1,033	0,302	0,987
miR-8059-5p	1,906	-0,044	0,104	-0,469	0,639	0,995
miR-8060-3p	2,373	-0,010	0,104	-0,100	0,920	0,995
miR-8061-3p	4,784	0,015	0,142	0,105	0,916	0,995
miR-8069-2-3p	7,261	0,093	0,188	0,507	0,612	0,995
miR-8072-5p	6,027	0,044	0,131	0,346	0,729	0,995
miR-8077-3p	56,563	0,191	0,261	0,742	0,458	0,995
miR-8078-5p	2,304	-0,061	0,116	-0,598	0,550	0,995
miR-8080-3p	1,465	0,079	0,104	1,440	0,150	0,894
miR-8085-3p	1,515	0,082	0,104	1,705	0,088	0,892
miR-8086-3p	12,178	0,084	0,206	0,413	0,679	0,995
miR-8485-5p	6,728	0,228	0,175	1,927	0,054	0,845
miR-873-3p	3,762	-0,024	0,139	-0,174	0,862	0,995
miR-874-3p	766,287	0,282	0,188	1,499	0,134	0,894
miR-874-5p	100,266	0,329	0,303	1,088	0,277	0,970
miR-875-5p	2,370	0,083	0,104	1,871	NA	NA

miR-877-3p	3,771	-0,058	0,146	-0,411	NA	NA
miR-877-5p	229,184	-0,366	0,266	-1,378	0,168	0,894
miR-885-3p	0,030	-0,020	0,096	-0,213	0,831	0,995
miR-885-5p	0,136	0,074	0,099	0,806	0,420	0,995
miR-887-3p	562,915	0,225	0,210	1,073	0,283	0,970
miR-887-5p	50,447	-0,383	0,316	-1,217	0,224	0,947
miR-9-1-3p	0,539	0,064	0,103	0,826	0,409	0,995
miR-9-1-5p	0,775	0,056	0,103	0,656	0,512	0,995
miR-921-5p	7,943	-0,026	0,194	-0,133	0,894	0,995
miR-922-3p	1,850	0,008	0,111	0,077	0,939	0,995
miR-92a-1-3p	306,422	-0,257	0,265	-0,968	0,333	0,995
miR-92a-1-5p	8,619	0,165	0,172	1,139	0,255	0,954
miR-92a-3p	1217,470	-0,134	0,241	-0,554	0,579	0,995
miR-92b-3p	3861,355	-0,097	0,255	-0,382	0,703	0,995
miR-92b-5p	45,027	-0,244	0,304	-0,805	0,421	0,995
miR-933-5p	0,737	-0,075	0,103	-1,154	0,249	0,954
miR-93-3p	319,126	-0,035	0,288	-0,123	0,902	0,995
miR-935-3p	86,879	-0,058	0,301	-0,194	0,846	0,995
miR-935-5p	25,408	0,052	0,229	0,228	0,820	0,995
miR-93-5p	335,985	0,162	0,253	0,641	0,522	0,995
miR-937-3p	3,643	-0,052	0,118	-0,477	0,633	0,995
miR-9-3p	5,251	-0,010	0,128	-0,076	0,940	0,995
miR-940	39,226	0,257	0,315	0,818	0,413	0,995
miR-940-3p	42,424	-0,048	0,316	-0,153	0,878	0,995
miR-941	186,620	0,327	0,278	1,179	0,238	0,954
miR-941-5-3p	24,423	0,094	0,288	0,325	0,745	0,995
miR-942-3p	2,991	-0,097	0,112	-2,076	0,038	0,829
miR-942-5p	12,828	-0,042	0,170	-0,250	0,802	0,995
miR-95-3p	111,050	-0,175	0,310	-0,566	0,571	0,995
miR-9-5p	11,611	-0,068	0,241	-0,283	0,777	0,995
miR-96-5p	1,801	0,048	0,104	0,523	0,601	0,995
miR-9718-3p	8,195	0,434	0,214	4,071	0,000	0,011
miR-98-3p	30,606	0,437	0,303	1,467	0,142	0,894
miR-98-5p	2573,549	0,171	0,207	0,826	0,409	0,995

miR-9899-3p	1,333	-0,036	0,104	-0,376	0,707	0,995
miR-9899-5p	11,315	-0,006	0,168	-0,034	0,973	0,995
miR-9901-3p	7,608	0,045	0,161	0,285	0,775	0,995
miR-9901-5p	12,404	0,065	0,162	0,413	0,680	0,995
miR-99a-3p	9,664	0,035	0,138	0,259	NA	NA
miR-99a-5p	9948,336	0,130	0,164	0,792	0,428	0,995
miR-99b-3p	100,397	0,019	0,301	0,062	0,951	0,995
miR-99b-5p	5261,866	0,029	0,147	0,198	0,843	0,995

Gene Targets

ARFGEF1
ABL1
ABL2
ABLIM1
ACACA
ACACB
ACAP3
ACIN1
ACTB
ACTG1
ACTN1
ACTN4
ACTR1B
ACTR2
ACTR3
ADCY10
ADCY2
ADCY5
ADCY6
ADCYAP1
ADCYAP1R1
ADIPOR1
ADIPOR2
ADORA2A
ADRA2B
ADRB1
ADRBK1
ADRBK2
AGAP1
AGAP2
AGAP3
AGPAT1
AGPAT2
AGPAT3
AGPAT4
AGPAT5
AHNAK
AKT1
AKT2
AKT3
ANAPC2
ANGPT4
ANK1
ANK2
ANK3
ANXA2
AP2A1
AP2B1
AP2M1

APC
APC2
APLN
APLNR
AR
ARAP1
ARAP3
ARF1
ARF3
ARF6
ARFGAP1
ARFGAP2
ARHGAP35
ARHGEF1
ARHGEF11
ARID1B
ARL8A
ARNT
ARNT2
ARPC1A
ARPC1B
ARPC2
ARRB1
ARRB2
ASAP2
ASAP3
ATF2
ATM
ATP1A1
ATP1B1
ATP2B2
ATP6AP1
ATP6V1H
ATR
AVPR1A
AXIN1
BAK1
BAX
BBC3
BCAR1
BCL2
BCL2L1
BCL2L11
BCL2L2
BCR
BDNF
BECN1
BIRC3
BIRC5
BIRC7

BMI1
BMPR2
BOC
BRCA1
BRD7
CACNA1A
CACNA1B
CACNA1C
CACNA1D
CACNA1E
CACNA1H
CACNA1I
CACNB3
CACNG2
CACNG4
CACNG8
CALM1
CALM3
CALML3
CALML6
CAMK2B
CAMK2G
CAPZB
CASP3
CASP9
CAV3
CBL
CCDC6
CCN2
CCNA2
CCND1
CCND2
CCND3
CCNE2
CCNG1
CD44
CD63
CDC25B
CDC37
CDK2
CDK6
CDKN1A
CDKN1B
CEBPA
CFL1
CFL2
CHAD
CHD4
CHMP1A
CHMP3

CHMP4A
CHMP6
CHMP7
CHRM1
CLTB
CLTCL1
CNR1
COL1A1
COL2A1
COL4A1
COL6A1
COL6A2
COL6A6
COL9A2
COMP
CPSF1
CPSF7
CREB3L1
CREB3L2
CREB3L3
CREBBP
CRKL
CRTC2
CSF1
CSF1R
CSF2RB
CSF3
CSF3R
CSNK1A1
CSNK2A1
CSTF2
CSTF2T
CTBP1
CTBP2
CTGF
CTNNA1
CTNNA3
CTNND1
CTSL
CTTN
CUL3
CUL4A
CUL4B
CYCS
CYFIP1
CYTH1
CYTH2
CYTH3
CYTH4
DAPK1

DAPK2
DAZAP1
DCTN5
DDB2
DDIT4
DDX39B
DDX5
DGKD
DGKE
DGKG
DGKI
DGKQ
DGKZ
DIAPH1
DICER1
DLG1
DLG4
DLL4
DNM1
DNMT1
DOCK1
DPYSL2
DPYSL5
DRD2
DUSP1
DUSP2
DUSP3
DUSP4
DUSP5
DUSP7
DUSP8
DUSP9
DVL1
DVL3
DYNC1H1
DYNC1LI2
DYNLL2
DYNLT3
E2F1
E2F2
EFNA1
EFNA2
EFNA4
EFNA5
EFNB1
EFNB3
EGFR
EGLN1
EHD1
EHD2

EHD3
EHD4
EIF4B
EIF4E
EIF4E2
EIF4EBP1
ELAVL1
ELK1
EP300
EPAS1
EPHA2
EPHA4
EPHA7
EPHA8
EPHB2
EPHB3
EPHB4
EPHB6
EPN1
EPN2
EPOR
ERBB2
ERBB3
ERBB4
EREG
ERG
ETS1
EVL
EXOC2
EXOC7
EZR
F2RL3
FASN
FBXW11
FCER1G
FGF1
FGF10
FGF17
FGF19
FGF2
FGF22
FGF3
FGF7
FGF9
FGFR1
FGFR2
FGFR3
FLNA
FLNB
FLOT2

FLT1
FN1
FNBP1
FOXO1
FOXO3
FOXO4
FOXP1
FRAT1
FRAT2
FRS2
FYCO1
FZD10
FZD2
FZD5
FZD7
FZD8
G6PC
G6PC3
GAB1
GAB2
GABARAPL1
GADD45G
GAPDH
GAS6
GBF1
GCK
GDF7
GHR
GIT1
GLE1
GLI2
GLI3
GLMN
GLS
GNA12
GNA13
GNAI2
GNAI3
GNAO1
GNAS
GNB1
GNB2
GNB3
GNB4
GNB5
GNG11
GNG13
GNG2
GNG4
GNG7

GPC1
GRB2
GRIA1
GRIN2A
GRIN2B
GRIN2D
GRK1
GRK5
GRK6
GRM1
GRM2
GRM4
GRM5
GSPT1
GSTM2
GSTM4
GSTM5
GSTO2
GUCY1A2
GUCY1A3
H3-3B
H3C15
HBS1L
HDAC4
HDAC5
HERC2
HERC3
HEY1
HEYL
HGS
HHIP
HIF1A
HK2
HKDC1
HLA-A
HLA-B
HLA-C
HLA-E
HLA-G
HMOX1
HNRNPK
HOXD10
HPSE2
HRAS
HSP90AB1
HSP90B1
HSP90AA1
HSPA1B
HSPA2
HSPA6

HSPG2
IFNAR2
IGF1
IGF1R
IGF2
IGF2BP1
IGF2R
IGFBP3
IKBKB
IL12RB2
IL15
IL15RA
IL1B
IL1R1
IL2RB
IL3RA
IL6R
IL6ST
IL7R
INPP5A
INPPL1
INSR
INSRR
IQGAP1
IQSEC2
IQSEC3
IRAK1
IRF1
IRF3
IRS2
IRS4
IST1
ITCH
ITGA1
ITGA10
ITGA11
ITGA3
ITGA5
ITGA6
ITGAV
ITGB1
ITGB4
ITGB6
ITGB8
JAG1
JAK3
JUN
JUND
KCNJ3
KCNJ5

KCNJ6
KCNJ9
KCNMA1
KEAP1
KIF5A
KIF5B
KIF5C
KIT
KIAA1033
KLF2
KLK3
KPNA4
KRAS
KSR1
KSR2
L1CAM
LAMA4
LAMA5
LAMB2
LAMC1
LAMC3
LAT
LATS1
LDHA
LDLR
LDLRAP1
LEF1
LIMK1
LIMK2
LLGL1
LPAR2
LPAR3
LRIG2
M6PR
MAGI1
MAML2
MAP2K1
MAP2K2
MAP2K3
MAP2K7
MAP3K3
MAP3K6
MAP3K7
MAP4K2
MAP4K4
MAPK1
MAPK13
MAPK3
MAPK7
MAPK8IP1

MAPK8IP2
MAPK8IP3
MAPKAPK2
MAPKAPK3
MAPT
MARCKS
MAX
MCL1
MDM2
MED12L
MED13
MED13L
MED16
MED17
MEF2A
MEF2B
MEF2C
MEF2D
MET
MID1
MITF
MKNK2
MLST8
MMP1
MMP16
MMP2
MPO
MRAS
MS4A2
MSH6
MSI2
MSN
MTNR1B
MTOR
MVB12B
MYD88
MYL12A
MYL9
MYLK
MYLK2
MYLPF
NCK1
NCKAP1
NCOA2
NCOA3
NCOA4
NECTIN2
NEDD4L
NF1
NFATC1

NFATC2
NFATC3
NFATC4
NFKB2
NFX1
NGFR
NOS1
NOS1AP
NOTCH1
NOTCH2
NOTCH3
NRAS
NRG1
NRG2
NRP1
NTN1
NTNG1
NTNG2
NTRK2
NUDT16L1
NUDT21
OSM
OSMR
PABPC1
PABPC4
PABPN1
PAK3
PAK4
PAK6
PAPOLA
PARD3
PARD6B
PARVA
PARVG
PAX8
PBRM1
PCK1
PDE3A
PDE3B
PDGFRA
PDGFRB
PDK1
PDPK1
PER1
PER2
PER3
PFKP
PFN1
PFN2
PGAM1

PHKB
PHKG2
PHLPP1
PHLPP2
PIK3C2A
PIK3C2B
PIK3C3
PIK3CD
PIK3R1
PIK3R3
PIK3R5
PIM2
PIP5K1C
PIP5KL1
PKLR
PKM
PKN3
PLA2G2C
PLA2G2F
PLA2G3
PLA2G4E
PLA2G4F
PLA2G6
PLAUR
PLCB1
PLCB3
PLCG1
PLD1
PLD2
PLEKHG5
PLIN1
PLXNA1
PLXNA2
PLXNA3
PLXNA4
PLXNB1
PLXNB2
PLXNB3
PML
PNN
POLK
PPAP2B
PPAP2C
PPARD
PPARG
PPIL2
PPP1CA
PPP1CB
PPP1CC
PPP1R12A

PPP1R12B
PPP1R12C
PPP1R3E
PPP2CB
PPP2R1A
PPP2R2A
PPP2R2C
PPP2R5A
PPP2R5B
PPP2R5D
PPP2R5E
PPP3R1
PPP3R2
PPP5C
PRKAB2
PRKACA
PRKACB
PRKACG
PRKAG1
PRKAR1B
PRKAR2A
PRKCA
PRKCE
PRKCI
PRKCZ
PRKD2
PRKG1
PRKAA1
PSD3
PSD4
PTCH1
PTGER1
PTGS2
PTK2B
PTPN11
PTPN5
PTPN7
PTPRF
PXN
PYGB
PYGM
RAB11A
RAB11B
RAB11FIP1
RAB11FIP3
RAB11FIP4
RAB11FIP5
RAB14
RAB22A
RAB35

RAB5A
RAB5B
RAB7A
RAB8A
RABEP1
RAC2
RAF1
RALBP1
RALGDS
RAP1GAP
RAPGEF1
RAPGEF3
RAPGEF5
RARA
RASA3
RASA4
RASAL2
RASD1
RASGRF1
RASGRF2
RASGRP3
RB1CC1
RBPJ
RCHY1
RELA
RET
RGL2
RGMA
RGS14
RHOA
RHOB
RHOQ
RNF103-CHMP3
RNF41
RNMT
RNPS1
ROCK2
RPS6
RPS6KA1
RPS6KA3
RPS6KA4
RPS6KA5
RPS6KA6
RPS6KB1
RPTOR
RUNX1
RUNX1T1
RXRA
RXRB
SDC1

SDC4
SEMA3A
SEMA3F
SEMA4A
SEMA4C
SEMA4D
SEMA4F
SEMA4G
SEMA5B
SEMA6A
SEMA6B
SEMA7A
SEPTIN11
SEPTIN8
SERPINE1
SESN1
SESN3
SGK2
SH3GL1
SH3GL2
SH3GL3
SH3GLB2
SHC1
SHC3
SHH
SIN3A
SIPA1L3
SIRT6
SLC16A10
SLC1A5
SLC25A6
SLC2A1
SLC2A4
SLC45A3
SLC7A5
SLC8A1
SLC8A2
SLC9A1
SLIT1
SLIT2
SLIT3
SMAD3
SMAP1
SMARCB1
SMARCD1
SMARCD2
SMARCE1
SMG1
SMO
SMURF1

SMURF2
SNAI2
SNX1
SNX18
SNX2
SOCS2
SOCS3
SOCS4
SORBS1
SOS1
SOX4
SP1
SPHK2
SPINT1
SPRY2
SQSTM1
SRC
SREBF1
SRF
SRGAP1
SRGAP3
SSH1
SSH2
SSH3
STAMBP
STAT1
STAT2
STAT3
STAT5B
STEAP3
STK11
STK4
STMN1
SUFU
SYK
SYNGAP1
TAB1
TAB2
TAOK1
TAOK2
TARDBP
TCF7
TCF7L1
TCF7L2
TEAD1
TEAD2
TERT
TFAP4
TFRC
TGFA

TGFBR2
THBS1
THBS2
THEM4
THRB
TIMP3
TJP1
TLN1
TLN2
TMPRSS2
TNC
TNF
TNFRSF10B
TNIP1
TNR
TNXB
TP53
TPM3
TPR
TRADD
TRAF1
TRAF2
TRAF3
TRAF4
TRAF6
TRIM71
TRIP12
TRPC5
TUBB
TUBB4B
TUBG1
TWIST2
TXNRD2
UBE2A
UBE2D1
UBE2D3
UBE2D4
UBE2E2
UBE2G1
UBE2G2
UBE2H
UBE2L3
UBE2O
UBE2Q1
UBE2Q2
UBE2W
UBE2Z
UBE3C
UBE4A
ULK1

UNC5A
UNC5B
UNC5C
UNC5D
VAV2
VAV3
VEGFA
VEGFB
VEGFC
VPS26B
VPS37A
VPS37C
VPS37D
VPS4A
VTA1
VTN
WASF2
WASL
WIPF2
WNT10B
WNT11
WNT2B
WNT3
WNT3A
WNT5A
WNT7A
WNT7B
WNT9A
WNT9B
XIAP
YAP1
YWHAB
YWHAG
YWHAH
YWHAZ
ZBTB16
ZBTB17
ZEB1
ZEB2
ZFYVE27
ZMAT3

```
#Installation of packgaes
install.packages("readxl")

if (!requireNamespace("BiocManager", quietly = TRUE))
  install.packages("BiocManager")
BiocManager::install("clusterProfiler")
BiocManager::install("org.Hs.eg.db")
BiocManager::install("DOSE")
BiocManager::install("multiMiR")
BiocManager::install("enrichplot")
BiocManager::install("ggalluvial")
BiocManager::install("scales")
BiocManager::install("RColorBrewer")
BiocManager::install("tidyr")
BiocManager::install("dplyr")
BiocManager::install("shadowtext")


# Load the packages
library("readxl")
library(clusterProfiler)
library(org.Hs.eg.db)
library(DOSE)
library(multiMiR)
library(enrichplot)
library(ggplot2)
library(tidyverse)
library(pathview)
library(ggalluvial)
library(scales)
library(RColorBrewer)
```

```
library(tidyr)
library(dplyr)
library(shadowtext)



#Read data from excel files
miRNA_data <- read_excel("C:/R_workplace/miRNA/miRNA_ver_1.xlsx")

#Extracting all miRNA names
all_miRNA <- miRNA_data[, "microRNA"]

#Checking information of multimiR package's databases
db.info = multimir_dbInfo()
db.info

validated_tables()

# Query the multiMiR database using all
all_results <- get_multimir(org     = 'hsa',
                    mirna   = all_miRNA,
                    table   = 'validated',
                    summary = TRUE)

head(all_results@data)

# Extracting the list of target genes and delete the repeated genes
all <- unique(all_results@data$target_symbol)

# Convert target symbol to Entrez IDs
# The "fromType" argument here is the way to test which method will map the maximum of gene IDs
all_ids <- bitr(all,
```

```r
            fromType = "ALIAS",
            toType = "ENTREZID",
            OrgDb = org.Hs.eg.db)

##Overrepresentation Analysis
### 1. GO enrichment analysis
  ####MF、CC、BP combination：
  all_GO_all <- enrichGO(gene = all_ids$ENTREZID,
                   OrgDb = org.Hs.eg.db,
                   ont = "ALL",
                   # molecular function (MF),
                   # biological process (BP),
                   # and cellular component (CC),
                   # "ALL" for all 3.
                   pAdjustMethod = "BH",
                   pvalueCutoff = 0.05,
                   qvalueCutoff = 0.05,
                   readable = TRUE)
  ####Extract result：
  GO_all_result <- all_GO_all@result

  ####3 ontology take top30 sorted based on p.adjust：
  all_result <- arrange(GO_all_result,p.adjust) #default ascending

  ####Take top30 terms：
  all <- all_result[1:30,]

  ####Setting factor：
  all$term <- factor(all$Description,levels = rev(all$Description))

  ####Setting colors for labels on y-axis for：
```

```
col_function <- function(x){
  col <- rep("black", length(x))
  BP <- which(x %in% c("BP"))
  CC <- which(x %in% c("CC"))
  MF <- which(x %in% c("MF"))
  col[BP] <- "#fc4d26"
  col[CC] <- "#1792c1"
  col[MF] <- "#3fad5d"
  col
}

y_text_color <- col_function(all$ONTOLOGY)

####Go dotplot
####Calculate Rich factor=GeneRatio/BgRatio
####"gene ratio" means the number of genes enriched in this term /total gene number
####"BgRatio" means the number of background genes enriched in this term /total background
gene number
rf<- apply(all,1,function(x){
  GeneRatio <- eval(parse(text = x["GeneRatio"]))
  BgRatio <- eval(parse(text = x["BgRatio"]))
  RF<- round(GeneRatio/BgRatio,2)
  RF
})
all$Rich_Factor <- rf


####Set theme
mytheme_dot <- theme(axis.ticks.y = element_blank(), #Delete y axis markings

          axis.title = element_text(size = 8),
```

```
                axis.text = element_text(size = 8),
                plot.title = element_text(size = 10,
                                          hjust = 0.5,
                                          face = "bold"),
                legend.title = element_text(size = 10),
                legend.text = element_text(size = 10)
                )

####Plot GoDot
ggplot(data = all,
       aes(x = reorder(term, Rich_Factor),
           y = Rich_Factor)) +
  geom_point(aes(size = Count, color = -log10(p.adjust))) + # setting the size and color of the dot
  coord_flip() + #reverse x and y
  labs(x = "Description",
       y = "Rich Factor",
       title = paste0("Top30 of GO enrichment Dotplot"), # Set title
       size = "Gene Number") + # set legend size
  theme_bw() +
  mytheme_dot +
  theme(axis.text.y = element_text(color=y_text_color))

ggsave(filename = "GO_1st.tiff",
       path = "C:/R_workplace/miRNA",
       width = 10,
       height = 10,
       device='tiff',
       dpi=600)
```

###2. KEGG enrichment analysis

```
all_KEGG_1 <- enrichKEGG(all_ids$ENTREZID,
```

```
                    organism = 'hsa',
                    pAdjustMethod = "BH",
                    pvalueCutoff = 0.05,
                    qvalueCutoff = 0.05)


  head(all_KEGG_1)
  AK <- as.data.frame(all_KEGG_1)

  ####Output the enrichment terms result and manually annotate categorical BRITE hierarchy parent terms
  write.csv(AK, "C:/R_workplace/miRNA/KEGG_map.csv", row.names=FALSE)
  ####Read in the updated datasheet
  KG <- read_csv("C:/R_workplace/miRNA/KEGG_map_BRITE_BAR.csv")

  ####Visualisation - barplot

  #### Preview the graph
  ggplot(KG, aes(x = reorder(Description, Count), y = Count)) +
    geom_bar(stat="identity") +
    coord_flip()

  ####seperate data into different groups
  na_index <- which(is.na(KG$Count))

  group <- c(NA, rep(KG$Description[na_index[1]], na_index[2] - na_index[1] - 1),
          NA, rep(KG$Description[na_index[2]], na_index[3] - na_index[2] - 1),
          NA, rep(KG$Description[na_index[3]], na_index[4] - na_index[3] - 1),
          NA, rep(KG$Description[na_index[4]], na_index[5] - na_index[4] - 1),
          NA, rep(KG$Description[na_index[5]], na_index[6] - na_index[5] - 1),
          NA, rep(KG$Description[na_index[6]], nrow(KG) - na_index[6]))
```

```
KG$Group <- group
KG$Description <- factor(KG$Description, levels = rev(KG$Description))

table(KG$Group)
####Set colours of each terms
colors <- c("black", rep("#9dd1c9", na_index[2] - na_index[1] - 1),
      "black", rep("#f2b06f", na_index[3] - na_index[2] - 1),
      "black", rep("#bebbd7", na_index[4] - na_index[3] - 1),
      "black", rep("#eb8776", na_index[5] - na_index[4] - 1),
      "black", rep("#88afcf", na_index[6] - na_index[5] - 1),
      "black", rep("#f4b76e", nrow(KG) - na_index[6]))

#### set font of each terms
face <- c("bold", rep(NULL, na_index[2] - na_index[1] - 1),
     "bold", rep(NULL, na_index[3] - na_index[2] - 1),
     "bold", rep(NULL, na_index[4] - na_index[3] - 1),
     "bold", rep(NULL, na_index[5] - na_index[4] - 1),
     "bold", rep(NULL, na_index[6] - na_index[5] - 1),
     "bold", rep(NULL, nrow(KG) - na_index[6]))

####draw final plot
ggplot(KG, aes(Description, Count)) +

  geom_bar(aes(fill = Group), stat = "identity") + #barplot

  geom_text(aes(label= Count, y = Count + 2), size = 2) + #set labels

  scale_fill_manual(values = c("#9dd1c9",
                "#f2b06f",
                "#bebbd7",
```

```
                    "#eb8776",
                    "#88afcf",
                    "#f4b76e")) + #set colours

coord_flip() + #reverse x and y

theme_bw() + #set theme

ylab("Number of Gene") +
xlab("") + # set axis title

theme(legend.position = "none", #delete colour scale

    panel.grid.major.x = element_blank(),
    panel.grid.minor.x = element_blank(),
    panel.grid.major.y = element_line(linetype = "dashed"),
    panel.grid.minor.y = element_line(linetype = "dashed"), #change the grid

    axis.ticks.y = element_blank(), #Delete y axis markings

    axis.text.y = element_text(face = "bold",
                      color = rev(colors),
                      hjust = 0,
                      size = 8, #align to the left
                      lineheight = 2), #Set y axis markings
    plot.title = element_text(hjust = 0.5, size = 10),
    text = element_text(family = "Times" )
) +

ggtitle("KEGG Pathway Enrichment")
```

```
ggsave(filename = "KEGG_1st.tiff",
       path = "C:/R_workplace/miRNA",
       width = 10,
       height = 10,
       device='tiff',
       dpi=600)
```

##Sankey Summary

```
### 1. Read in the provided data
data <- readxl::read_excel("miR_Sankey.xlsx")


### 2. Prepare the data for the Sankey diagram
data$miRNA <- as.factor(data$miRNA)
data$Gene_Target <- as.factor(data$Gene_Target)
data$FinalBox <- as.factor(data$FinalBox)
data$Positive_or_negative <- as.factor(data$Positive_or_negative)


### 3. Get a diverging color palette.
### This creates a diverging color palette and sets up a color scale for the "-log2FC" values.
### The palette goes from red to blue, with a total of 100 colors.
color_pal <- colorRampPalette(brewer.pal(11, "RdBu"))(100)
color_scale <- scale_fill_gradientn(colors = rev(color_pal))
display.brewer.pal(11, "RdBu")


### 4. Plot the Sankey diagram for the colour of miRNA column


ggplot(data = data,
       aes(axis1 = miRNA, axis2 = Gene_Target, axis3 = FinalBox)) +


  geom_alluvium(aes(fill = log2FC)) + #The fill aesthetic of the geom_alluvium is set to the negative of log2FC (-log2FC) to invert the color for the links.
```

```
  geom_stratum(aes(fill = -log2FC)) + #The fill aesthetic of the geom_stratum is set to log2FC to color the miRNA tiles based on their expression level.

  # Replace geom_text with geom_shadowtext for labels on the strata
  geom_shadowtext(stat = "stratum",
                  size = 5,
                  fontface = "bold",
                  color = "#000000",  # Font color set to Deep Black
                  bg.colour = "white",  # White edge for the font
                  bg.r = 0.1,  # Radius of the edge color
                  aes(label = after_stat(stratum))) +

  labs(x = '', y = '', fill='Log2FC') + #Delete the x and y axis symbol

  # Replace annotate with shadowtext::geom_shadowtext for column names
  geom_shadowtext(data = data.frame(x = c(1, 2, 3), y = c(65, 65, 65),
                                    label = c("miR", "Gene Target", "Impact")),
                  aes(x = x, y = y, label = label),
                  size = 5,
                  fontface = "bold",
                  color = "#000000",
                  bg.colour = "white",
                  bg.r = 0.1,
                  inherit.aes = FALSE) +  # Set inherit.aes to FALSE

  color_scale +

  theme_minimal() +

  theme(legend.text = element_text(colour="black", size=10, face="bold"),
    panel.background = element_blank(),
```

```
          line = element_blank(),
          axis.text.y = element_blank(),
          axis.text.x = element_blank(),
          legend.position = "left") #Delete background grid and Move legend to the left side



      ggsave(filename = "sankey_part 1.tiff",
             path = "C:/R_workplace/miRNA",
             width = 15,
             height = 16,
             device='tiff',
             dpi=600)

    ### 5. Plot the Sankey diagram for the colour of Gene_Target column
      ggplot(data = data,
             aes(axis1 = miRNA, axis2 = Gene_Target, axis3 = FinalBox)) +

        geom_alluvium(aes(fill = log2FC)) + #The fill aesthetic of the geom_alluvium is set to the negative of log2FC (-log2FC) to invert the color for the links.

        geom_stratum(aes(fill = log2FC)) + #The fill aesthetic of the geom_stratum is set to log2FC to color the miRNA tiles based on their expression level.

        # Replace geom_text with geom_shadowtext for labels on the strata
        geom_shadowtext(stat = "stratum",
                        size = 5,
                        fontface = "bold",
                        color = "#000000",  # Font color set to Deep Black
                        bg.colour = "white",  # White edge for the font
                        bg.r = 0.1,  # Radius of the edge color
                        aes(label = after_stat(stratum))) +
```

```
  labs(x = '', y = '', fill='Log2FC') + #Delete the x and y axis symbol

  # Replace annotate with shadowtext::geom_shadowtext for column names
  geom_shadowtext(data = data.frame(x = c(1, 2, 3), y = c(65, 65, 65),
                                    label = c("miR", "Gene Target", "Impact")),
                  aes(x = x, y = y, label = label),
                  size = 5,
                  fontface = "bold",
                  color = "#000000",
                  bg.colour = "white",
                  bg.r = 0.1,
                  inherit.aes = FALSE) +  # Set inherit.aes to FALSE

  color_scale +

  theme_minimal() +

  theme(legend.text = element_text(colour="black", size=10, face="bold"),
        panel.background = element_blank(),
        line = element_blank(),
        axis.text.y = element_blank(),
        axis.text.x = element_blank(),
        legend.position = "left")  #Delete background grid

ggsave(filename = "sankey_part 2.tiff",
       path = "C:/R_workplace/miRNA",
       width = 15,
       height = 16,
       device='tiff',
       dpi=600)
```

###6. Plot the Sankey diagram for the colour of Impact column

Assuming your 'Impact' column is within a dataframe called 'data'

```
data$FinalBox <- gsub("/", "\n", data$FinalBox, fixed = TRUE)  # Replace the first space with a line break

mycol <- c("#bebbd7", "#9dd1c9")

ggplot(data = data,
    aes(axis1 = miRNA, axis2 = Gene_Target, axis3 = FinalBox)) +

  geom_alluvium(lode.guidance = "forward",
        aes(fill = Positive_or_negative),
        aes.bind = "flows") + #The fill aesthetic of the geom_alluvium is set to the negative of log2FC (-log2FC) to invert the color for the links.

  geom_stratum(aes(fill = Positive_or_negative)) + #The fill aesthetic of the geom_stratum is set to log2FC to color the miRNA tiles based on their expression level.

  geom_text(stat = "stratum",
      size = 3.5,
      fontface = "bold",
      colour = "#000000",
      aes(label = after_stat(stratum))) +

  labs(x = '',
    y = '',
    fill='Log2FC') +  #Delete the x and y axis symbol

  # Replace annotate with shadowtext::geom_shadowtext for column names
  geom_shadowtext(data = data.frame(x = c(1, 2, 3), y = c(65, 65, 65),
                  label = c("miR", "Gene Target", "Impact")),
        aes(x = x, y = y, label = label),
```

```
              size = 5,
              fontface = "bold",
              color = "#000000",
              bg.colour = "white",
              bg.r = 0.1,
              inherit.aes = FALSE) +  # Set inherit.aes to FALSE

  scale_fill_manual(values = mycol) +

  theme_minimal() +

  theme(legend.text = element_text(colour="black", size=10, face="bold"),
       panel.background = element_blank(),
       line = element_blank(),
       axis.text.y = element_blank(),
       axis.text.x = element_blank(),
       legend.position = "left")  #Delete background grid

ggsave(filename = "sankey_part 3.tiff",
      path = "C:/R_workplace/miRNA",
      width = 15,
      height = 16,
      device='tiff',
      dpi=600)
```
